# Ewing sarcoma resistance to SP-2509 is not mediated through KDM1A/LSD1 mutation

**DOI:** 10.18632/oncotarget.26326

**Published:** 2018-11-23

**Authors:** Kathleen I. Pishas, Stephen L. Lessnick

**Affiliations:** ^1^ Cancer Therapeutics Laboratory, Discipline of Medicine, University of Adelaide, Adelaide, SA, Australia; ^2^ Center for Childhood Cancer and Blood Disorders, The Research Institute at Nationwide Children's Hospital, Columbus, OH, USA; ^3^ Division of Pediatric Hematology/Oncology/Bone Marrow Transplant, Ohio State University, Columbus, OH, USA

**Keywords:** Ewing sarcoma, LSD1, KDM1A, SP-2509

## Abstract

Ewing sarcoma is the second most common solid bone malignancy diagnosed in pediatric and young adolescent populations. Despite global co-operative efforts, outcomes for patients with relapsed and refractory disease remains obstinately poor. It has become increasingly clear that disruption of the epigenome as a result of alterations in epigenetic regulators, plays a pivotal role in tumorigenesis. As such, this study investigated Ewing sarcoma mechanisms of acquired resistance to the small molecule reversible lysine specific demethylase (*LSD1*/*KDM1A*) inhibitor SP-2509. Surprisingly, whole exome sequencing analysis of our generated A673 SP-2509 drug resistant cell line revealed an absence of mutations in *KDM1A*. Compared to parental counterparts, SP-2509 drug resistant cells demonstrated decreased anchorage independent growth capacity, enhanced sensitivity to the HDAC inhibitors vorinostat/entinostat and a distinct transcriptional profile that was enriched for extracellular matrix proteins. SP-2509 drug resistant cells also exhibited elevated expression levels of the multi-drug resistance genes *ABCB1*, *ABCC3*, and *ABBC5* and decreased expression of the transcriptional repressor *RCOR1*/*CoREST*. Following several months of SP-2509 withdrawal, low level SP-2509 resistance was still apparent (6.3 fold increase in IC_50_), with drug resistant cell populations maintaining their distinct transcriptional profile. Furthermore, compared to parental cells, washout drug resistant lines displayed equal sensitivity to the standard Ewing sarcoma chemotherapeutic agent's vincristine and doxorubicin. Together these findings indicate that resistance to SP-2509 is not fully reversible or driven by direct mutation in *KDM1A*.

## INTRODUCTION

Ewing sarcoma is a rare malignancy of bone, which predominantly afflicts children and young adolescent populations. The unifying genetic trait of this highly aggressive cancer is a reciprocal chromosomal translocation that fuses the *EWSR1* gene with members of the ETS family of transcription factors, most commonly *FLI1* (85% of cases) [1]. The resulting fusion protein is responsible for oncogene activation, inhibition of tumor suppression, chromatin remodeling and epigenomic reprogramming [2–4]. Despite our growing appreciation of the molecular underpinnings that drive this tumor, standard first line treatment still relies on traditional intensive induction chemotherapy (doxorubicin, vincristine, cyclophosphamide, etoposide and ifosfamide) followed by local control with surgery and/or radiotherapy and consolidation chemotherapy. Although this strategy has proven to be efficacious for the treatment of localized disease, long-term survival rates for patients with metastatic or relapsed Ewing sarcoma remains unacceptably low (< 30%) [5]. Development of resistance to chemotherapy and associated toxicities remains the main cause of treatment failure, with global co-operative efforts investigating dose intensification and interval compression failing to improve survival rates thus far. As metastatic tumors are often refractory to conventional chemotherapy and irradiation, this underscores the formidable challenge to develop and incorporate novel targeted agents to combat this nefarious pediatric cancer.

It has become increasingly clear that disruption of the epigenome as a result of alterations in epigenetic regulators is a fundamental driver mechanism in cancer. Indeed, histone methylation is a major determinant of chromatin structure and function and has shown to play critical roles in transcriptional regulation and genomic stability [6]. This is particularly pertinent for Ewing sarcoma, as recent high throughput screening efforts have shown that this malignancy possesses one of the lowest mutation rates amongst all cancers (0.15 mutations/Mb) [7, 8], yielding a paucity of pharmacologically actionable mutations. Several studies have reported overexpression of lysine specific demethylase 1A (*LSD1)*, also known as *KDM1A/BHC110* which regulates chromatin states through the removal of mono and dimethyl groups (H3K4 or H3K9) in both Ewing sarcoma cell lines and tumors [9, 10]. We recently demonstrated that Ewing sarcoma cell lines are highly susceptible to KDM1A blockade with the small molecule inhibitor SP-2509. This non-competitive reversible inhibitor induces apoptotic responses in Ewing sarcoma cell lines through engagement of the endoplasmic reticulum stress response, reverses the EWS/ETS transcriptional signature, impairs several EWS/ETS-associated oncogenic phenotypes, and shows single-agent efficacy in multiple xenograft models of Ewing sarcoma [10, 11]. As clinical formulations of SP-2509 (Seclidemstat, *Salarius Pharmaceuticals*) entered phase I clinical testing for Ewing sarcoma patients in 2018, it is imperative that possible mechanisms underlying resistance are elucidated.

As we strive to prolong patient survival while minimizing toxicity, the advent of targeted therapy for the treatment of cancer has significantly added to our armamentarium. Unfortunately, both chemotherapy and molecularly targeted approaches share the overarching limitation of the emergence of drug resistance, which prevents these drugs from eliciting lasting clinical benefit. Cancer cells employ numerous intrinsic (innate) and extrinsic (acquired) avenues to promote drug resistance. These include increasing drug efflux, enzymatic modification/inactivation of the drug, alteration or mutation of the drug target, drug detoxification, overexpression of proteins that compensate for the loss of the drug target, and activation of redundant biological feedback mechanisms [12]. Indeed, several studies have shown that Ewing sarcoma cell lines and tumors basally express high levels of the drug-efflux proteins *MDR1* (P-glycoprotein), *MRP1*, *ABCB1* and *ABCG2* [13–15].

One key aspect towards realizing the potential of targeted therapies is a better understanding of the intrinsic or acquired resistance mechanisms that limit their efficacy. As high level intrinsic resistance to SP-2509 was not observed in our Ewing sarcoma cell line panel (*n* = 17), this study generated a SP-2509 drug resistant Ewing sarcoma cell line to identify acquired SP-2509 resistance mechanisms. Interestingly we show that direct mutation of *KDM1A* does not contribute to SP-2509 drug resistance, instead drug resistant cells develop a non-reversible distinct transcriptional profile characterized by enhanced expression of extracellular matrix proteins. Furthermore, we show that SP-2509 drug resistant cells are highly sensitive to the HDAC inhibitors entinostat and vorinostat, possibly providing a second line therapeutic approach.

## RESULTS

### SP-2509 drug resistant A673 cells display altered proliferative and anchorage independent growth

**Figure 1:**
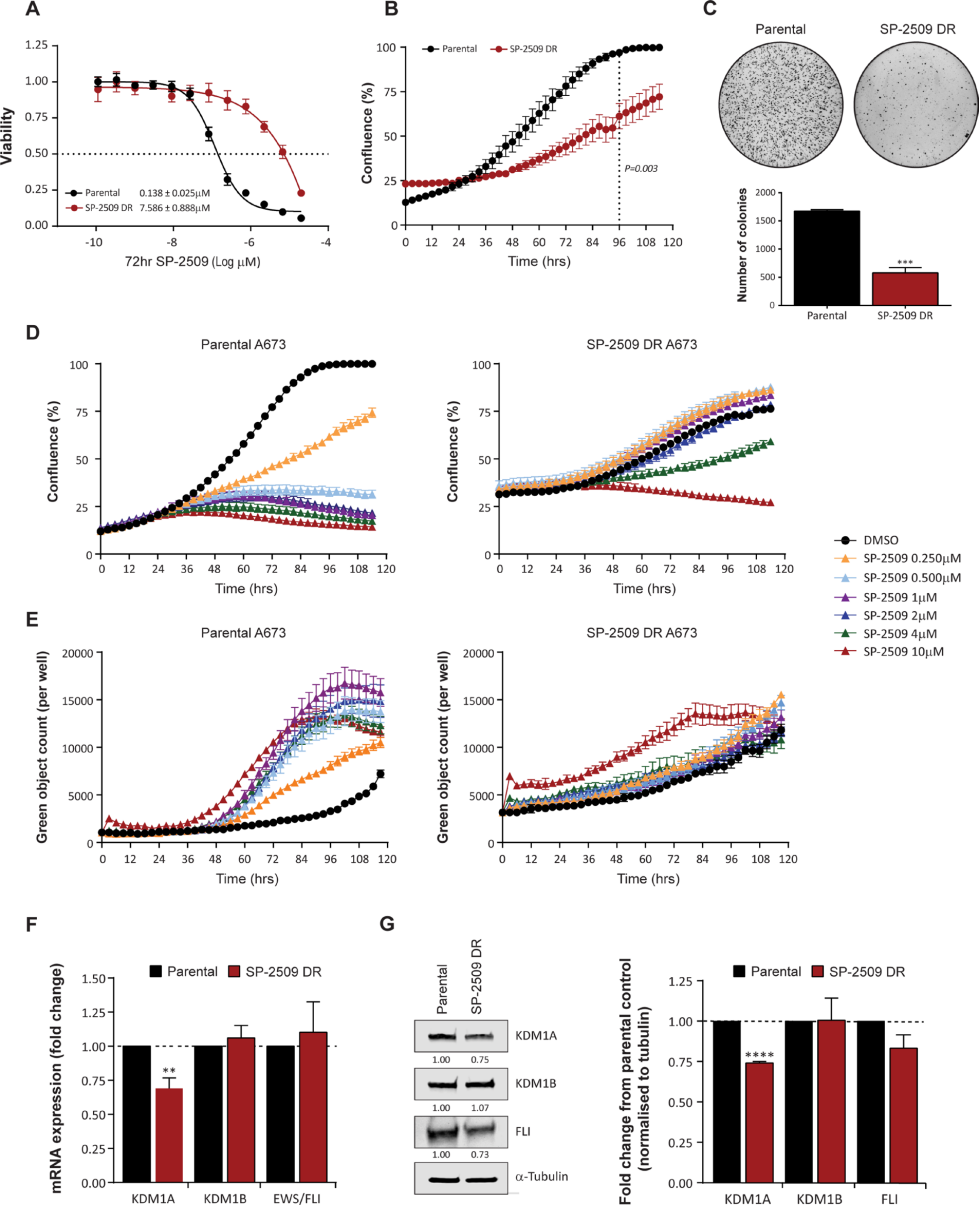
SP-2509 drug resistant A673 cells display reduced proliferative and anchorage independent growth (**A**) Sensitivity of parental and SP-2509 drug resistant (DR) A673 cells to SP-2509. Cell viability assessed through CellTiter Glo analysis, 72 hrs after treatment. (**B**) 120 hr IncuCyte proliferative growth analysis of parental and SP-2509 DR A673 cells. (**C**) Anchorage independent growth of parental and SP-2509 DR A673 cells determined through soft agar assays. Quantification of the average number of colonies per plate is also depicted. IncuCyte proliferative growth (**D**) and Caspase 3/7 induction (**E**) of parental and SP-2509 DR A673 cells treated with the indicated concentrations of SP-2509 or vehicle control (DMSO) for 120 hrs. Basal mRNA (**F**) and (**G**) protein levels of KDM1A, KDM1B and EWS/FLI from parental and SP-2509 DR A673 cells. All data represents mean ± SEM from three independent experiments. Asterisks denote statistical significance ^**^P < 0.01, ^***^P,0.001, ^****^P < 0.0001.

Drug resistance in cancer frequently emerges during treatment, particularly with novel targeted therapies designed to inhibit specific molecules. We previously demonstrated that Ewing sarcoma cell lines are exquisitely sensitive to SP-2509, a reversible, non-competitive small molecule inhibitor of *KDM1A*. As innate resistance to SP-2509 was not observed in our Ewing sarcoma cell line cohort (*n* = 17) [10], we attempted to generate four drug resistant (DR) cell lines (A673, TC252, TC32, TTC-466) through chronic exposure to increasing concentrations of SP-2509. Of these lines, A673 cells were the only line that could stably grow in SP-2509 concentrations exceeding 2 μM and hence were chosen for further exploration. Indeed, following prolonged continuous treatment for seven months, a 55.0 fold increase in SP-2509 concentration was required to reduce the viability of generated A673 SP-2509 DR cells compared to parental controls (IC_50_: 0.138 μM versus 7.586 μM) by 50% (Figure 1A). In addition, compared to parental controls, A673 SP-2509 DR cells demonstrated significantly reduced proliferative capacity (Figure 1B) and anchorage independent growth (2.9 fold decrease in colony number, *P = 0.0003*) (Figure 1C). Treatment of A673 parental cells with doses of SP-2509 as low as 0.250 μM significantly reduced their proliferative capacity and induced apoptosis as evidenced through caspase 3/7 induction. In contrast, diminished proliferative capacity of SP-2509 DR cells was only observed at SP-2509 concentrations exceeding 4 μM, with apoptotic cytotoxicity only observed at 10 μM (Figure 1D, 1E). We previously showed that SP-2509 treatment significantly decreases both KDM1A mRNA and protein levels in Ewing sarcoma cells [10]. Correspondingly, A673 SP-2509 DR cells showed a 30.9% and 25.9% basal decrease in KDM1A mRNA and protein respectively. No significant changes in basal KDM1B/LSD2 and EWS/FLI expression were observed (Figure 1F, 1G).

### SP-2509 drug resistant A673 cells show enhanced sensitivity to HDAC inhibitors

**Figure 2:**
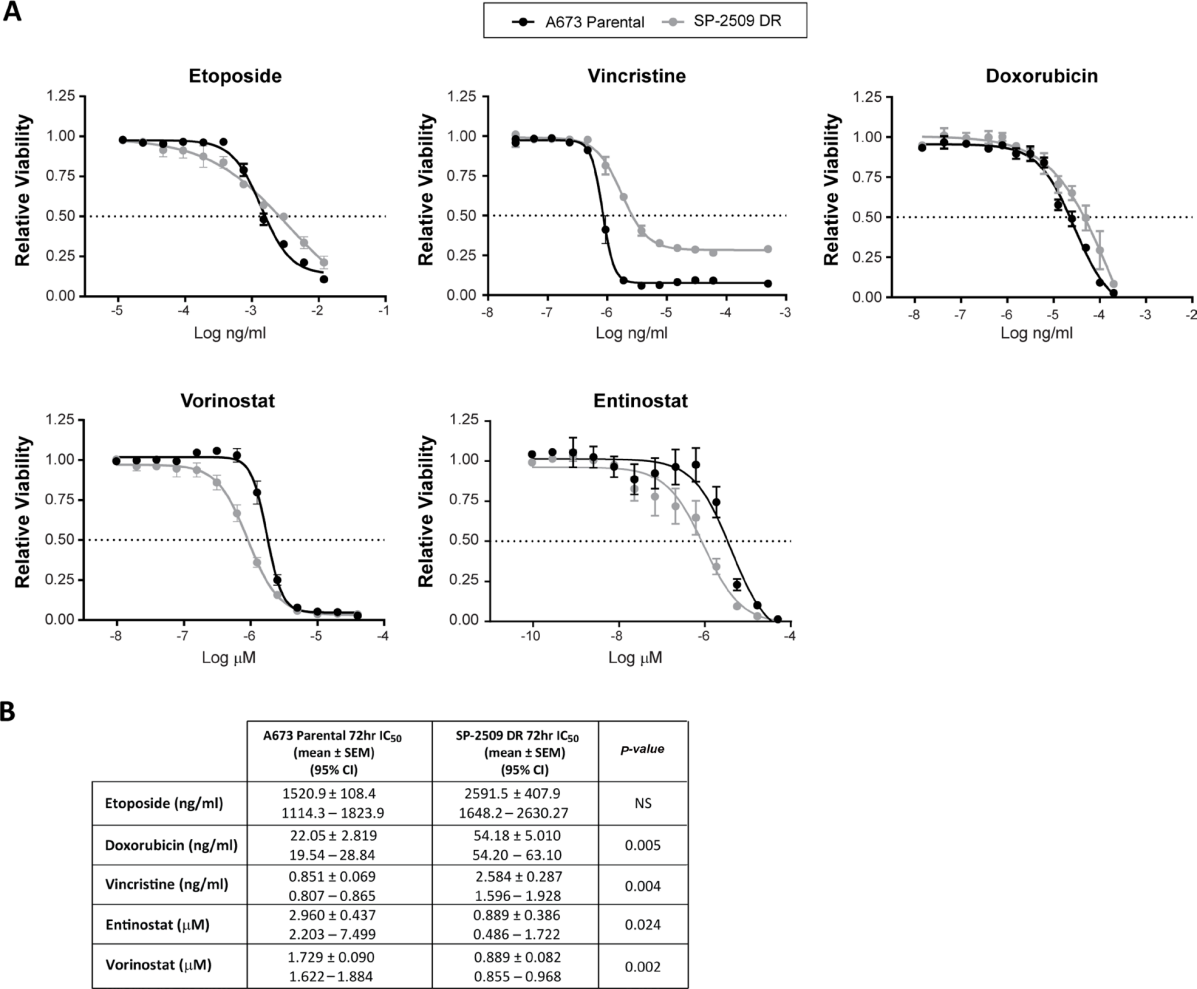
SP-2509 drug resistant A673 cells display enhanced sensitivity to HDAC inhibitors (**A**) Parental and SP-2509 DR A673 cells were treated with the indicated agents for 72 hrs. Cell viability was assessed through CellTiter Glo analysis. Data represents mean viability ± SEM from three independent experiments. (**B**) Mean IC_50_ ± SEM and 95% confidence interval for each agent.

We next investigated whether DR SP-2509 A673 cells display altered sensitivity to the standard Ewing sarcoma chemotherapeutic agents etoposide, vincristine, and doxorubicin. Following 72 hrs of drug exposure, SP-2509 DR A673 cells exhibited a 2.6 and 3.0 fold decreased sensitivity to doxorubicin (*P = 0.005*) and vincristine (*P = 0.004*) respectively, with sensitivity to etoposide maintained (Figure 2). We previously demonstrated that histone deacetylases HDAC2/3 are directly recruited by EWS/FLI to mediate transcriptional repression, with vorinostat treatment of Ewing sarcoma cell lines blocking EWS/FLI–mediated transcriptional repression but not activation [16]. As such we examined the sensitivity of SP-2509 DR cells to the HDAC inhibitors entinostat and vorinostat. Surprisingly, DR cells demonstrated enhanced sensitivity to both entinostat (*P = 0.024*) and vorinostat (*P = 0.002*), 4.3 and 1.94 fold increase respectively. This suggests that HDAC inhibitors could be used to overcome SP-2509 resistant cell populations.

### Resistance to SP-2509 is not mediated through mutation in *KDM1A*

Resistance to targeted agents primarily occurs through direct mutation of the drug target itself, thereby preventing binding of the drug to its intended target. To address whether resistance to SP-2509 is a direct consequence of acquired mutations in *KDM1A*, sanger sequencing of all 19 exons of *KDM1A* was performed. Surprisingly no mutations in *KDM1A* were observed. To establish whether resistance can be attributed to mutation in other key *KDM1A* interacting partners, A673 SP-2509 DR and parental cells were subjected to 250X whole exome sequencing (WES). Twenty-three mutations were identified specifically in the SP-2509 DR population, with no aberrations in *KDM1A* or its interacting partners (CoREST, NuRD, HDAC) observed (Table 1). The majority of mutations (20/23) occurred within coding sequences, with the remaining 3 mutations located within splice site or extended intronic splice regions. It must be noted that only 1/23 mutations identified occurred in greater than 50% of the cell population, highlighting that resistance to SP-2509 is not mediated through mutational means. The most prevalent mutation (88.4% of total population) was recorded in *MRPL45* (mitochondrial ribosomal protein L45) (Glu53^*^) which encodes the ribosomal 39S subunit protein. A second stop-gain mutation in *MRPS10* (mitochondrial ribosomal protein S10) (Glu146^*^) which encodes the 28S subunit protein was also observed in 35.6% of the SP-2509 DR cell population.

**Table 1:** Mutations identified in SP-2509 drug resistant A673 cells

Rank	Position	Gene		Loc in gene	Effect	HGVS	Population (%)
1	17q12	*MRPL45*	mitochondrial ribosomal protein L45	CS	Stopgain	p.Glu53^*^	88.4%
2	15q13.1	*OCA2*	OCA2 melanosomal transmembrane protein	splice_site		c.1843-1G>T	45.2%
3	9q34.11	*HMCN2*	hemicentin 2	CS	NS-SNV	p.Ala352Glu	45.1%
4	1q25.1	*SERPINC1*	serpin family C member 1	CS	NS-SNV	p.Ser169Ala	44.3%
5	15q11.2	*MAGEL2*	MAGE family member	CS	NS-SNV	p.Thr1158Pro	42.9%
6	1p34.3	*GJB4*	gap junction protein beta 4	CS	NS-SNV	p.Asp118Glu	42.2%
7	1p36.12	*ALPL*	alkaline phosphatase, liver/bone/kidney	CS	NS-SNV	p.Ala503Thr	39.9%
8	1p36.12	*E2F2*	E2F transcription factor 2	EISR		c.738-9C>A	39.4%
9	5p15.31	*SEMA5A*	semaphorin 5A	CS	NS-SNV	p.Asp720Glu	39.1%
10	6p12.3	*PKHD1*	PKHD1, fibrocystin/polyductin	CS	NS-SNV	p.Gly3809Cys	39.0%
11	19q13.12	*ZNF781*	zinc finger protein 781	CS	Startloss		38.7%
12	16p11.2	*APOBR*	apolipoprotein B receptor	CS	NS-SNV	p.Leu409Arg	38.7%
13	8p21.3	*SH2D4A*	SH2 domain containing 4A	CS	NS-SNV	p.Pro89Thr	38.4%
14	17q25.3	*CSNK1D*	casein kinase 1 delta	CS	NS-SNV	p.Arg279Cys	38.3%
15	1p13.3	*LAMTOR5*	late endosomal/lysosomal adaptor,	CS	NS-SNV	p.Ala135Asp	36.5%
16	11q23.3	*CBL*	Cbl proto-oncogene	EISR		c.444-9C>A	36.0%
17	6p21.1	*MRPS10*	mitochondrial ribosomal protein S10	CS	Stopgain	p.Glu146^*^	35.6%
18	12q24.13	*TPCN1*	two pore segment channel 1	CS	NS-SNV	p.Gly779Val	31.4%
19	Xq11.2	*MTMR8*	myotubularin related protein 8	CS	NS-SNV	Arg541Leu	29.9%
20	6p21.32	*HLA-DQA1*	major histocompatibility complex, class II, DQ alpha 1	CS	NS-SNV	p.Ser119Tyr	28.3%
21	11p15.5	*IRF7*	interferon regulatory factor 7	CS	NS-SNV	p.Arg109Leu	27.7%
22	Xq12	*HEPH*	hephaestin	CS	NS-SNV	p.Tyr789Cys	11.1%
23	14q32.33	*AHNAK2*	AHNAK nucleoprotein 2	CS	NS-SNV	p.Lys89Asn	11.0%

Abbreviations: CS: Coding sequence, EISR: Extended intronic splice region, NS-SNV: Non-synonymous single nucleotide variant.

To confirm that these mutations were not a direct consequence of continuous long-term passage, WES of parental A673 cells grown for 2 weeks versus 7 months was also evaluated. Fifty-one mutations were acquired during long term passage, with only one mutation in *C2CD2L* (Glu178Asp) common between A673 cells grown for an extended period and SP-2509 DR cells (Supplementary Table 1).

### SP-2509 drug resistant cells display a unique transcriptomic profile

**Figure 3:**
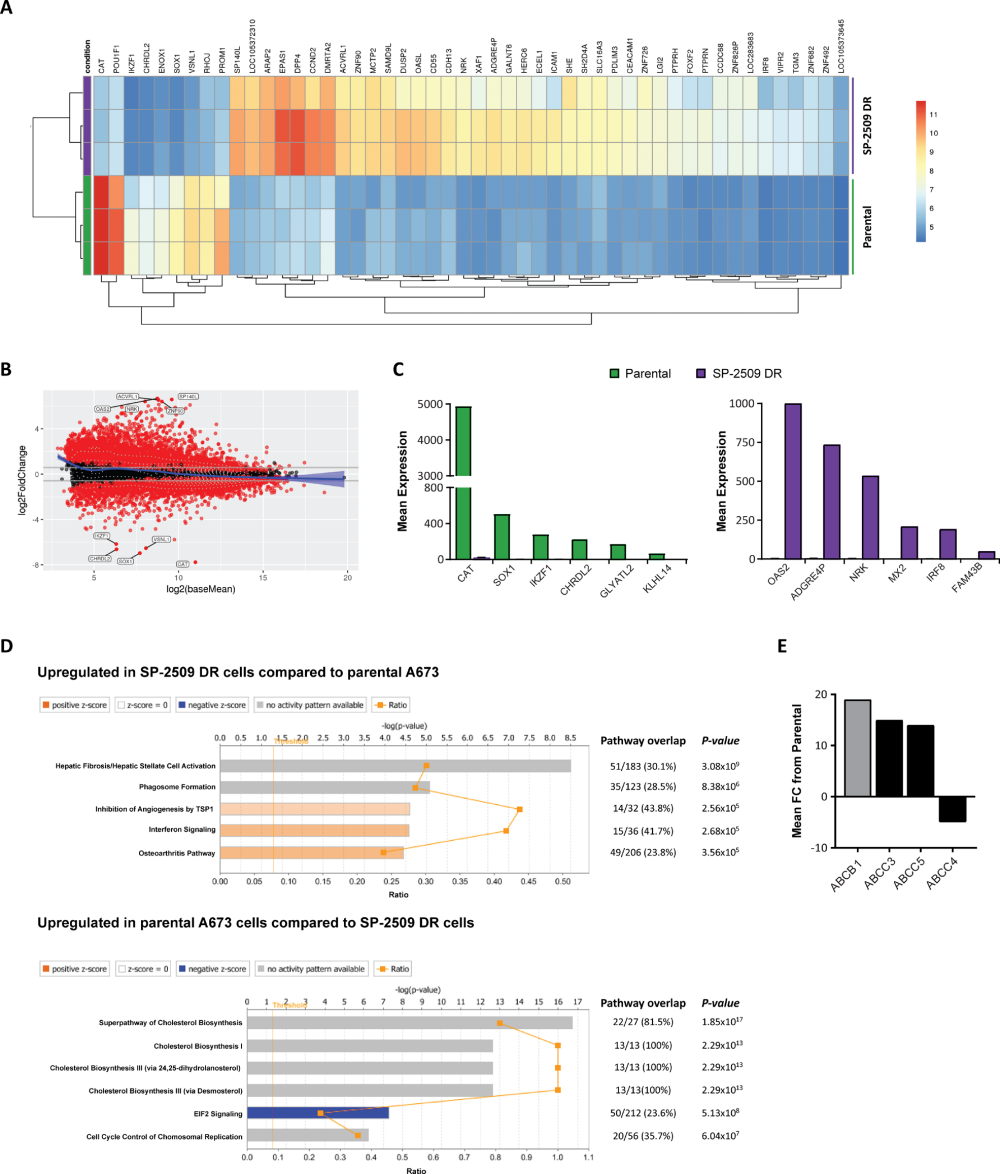
SP-2509 drug resistant cells show a distinct transcriptional profile (**A**) Unsupervised clustering analysis of the basal transcriptional profile of parental and SP-2509 DR A673 cells. The top 50 differentially expressed genes from RNA-Seq analysis are shown. (**B**) MvA plot showing differentially expressed genes (RNA-Seq) from parental and SP-2509 DR cells. X-axis denotes average expression level, with binary logarithm of fold-change shown on the y-axis. Horizontal grey bars show the fold change cutoff for significance (1.5). (**C**) Normalized mean expression of the top 6 highly expressed genes differentially expressed between parental A673 and SP-2509 DR cells. (**D**) IPA of genes significantly upregulated in SP-2509 DR cells versus parental cells and parental cells compared to SP-2509 DR cells (>1.5 fold). Percentage gene pathway overlap and *P*-value also depicted. (**E**) Average normalized expression of *ABCB1*, *ABCC3*, *ABCC5* and *ABCC4* in SP-2509 DR cells. Data represents fold change from parental A673 cells.

As resistance to SP-2509 was not mediated through mutation in *KDM1A*, we next addressed whether SP-2509 drug resistance is attributed to a specific transcriptional signature. Parental and SP-2509 DR A673 cells were submitted for RNA-Seq analysis with unsupervised hierarchical clustering revealing that SP-2509 DR cells do indeed display a unique profile distinct from their parental counterparts. In total, 3389 genes were significantly upregulated in our DR population (>1.5 fold) compared to parental cells with 2574 genes significantly upregulated in parental but not DR cell populations (Figure 3A). The top 5 protein coding genes significantly repressed (>270 fold reduction) in SP-2509 DR cells included *CHRDL2*, *SOX1*, *IKZF1*, *CAT*, and *GLYATL2*. Conversely, the top 5 genes significantly upregulated (>240 fold increase) in SP-2509 DR cells included *MX2*, *OAS2*, *IRF8*, *FAM43B* and *NRK*. (Figure 3B, 3C). Ingenuity Pathway Analysis (IPA) of the cohort of genes highly expressed only in SP-2509 DR cells revealed enrichment for the Hepatic Fibrosis pathway (*p* = 3.08 × 10^6^) which is affiliated with the accumulation of extracellular matrix proteins (Figure 3D). Out of the 183 genes associated with this pathway 51 (30.1%) were significantly modulated in DR cells including *ICAM1, VEGFC, COL6A3* and *COL1A.* Interestingly, Hepatic Fibrosis was the most common pathway associated with both *KDM1A* and EWS/FLI knockdown in A673 cells [10] demonstrating that SP-2509 DR cells mimic cells with KDM1A/EWS-FLI loss.

### SP-2509 does not engage the endoplasmic reticulum stress response in drug resistant cells

We previously determined that the mechanism of SP-2509 cytotoxic action is through engagement of the unfolded protein/endoplasmic reticulum (ER) stress response [10]. RNA-Seq analysis of parental and DR cells following SP-2509 treatment (2 μM, 48 hrs) confirmed our former findings, with 27 genes associated with these two cellular pathways significantly upregulated in parental A673 cells. In contrast only 2 of these 27 genes, *ATF4* and *CEBPG* were upregulated in SP-2509 DR cells following treatment. IPA of the cohort of induced genes modulated by SP-2509 in DR cells revealed enrichment for the following five pathways, netrin signaling (*P* = 3.52 × 10^5^), phenylalanine degradation IV (*P* = 2.84 × 10^5^), serine biosynthesis (*P* = 8.81 × 10^4^), AMPK signaling (*P* = 1.07 × 10^4^) and xenobiotic metabolism signaling (*P* = 1.44 × 10^3^) (Supplementary Figure 1). This suggests that SP-2509 is unable to induce cytotoxic effects in DR cells due to the loss of ER stress response engagement.

Overexpression of drug transporters such as P-glycoprotein (Pgp) also known as *MDR1/ABCB1*, and the MDR associated protein 1 (MRP) ATP-binding cassette (ABC) transporter family are well-established causes of multi-drug resistance. From our RNA-Seq analysis we next addressed whether SP-2509 drug resistance can be attributed to overexpression of these transporters. Basal expression of *ABCB1*, *ABCC3* and *ABBC5* was significantly higher in SP-2509 DR cells compared to parental A673 cells (19.2, 15.0 and 14.3 fold increase). In contrast, expression of *ABCC4* which is implicated in the transport of antiviral agents and endogenous molecules [17] was significantly decreased (4.5 fold) in SP-2509 DR cells (Figure 3E). Interestingly, in a panel of 6 Ewing sarcoma cell lines, high basal expression levels of *ABCC4* were associated with enhanced sensitivity to SP-2509 (*R*^2^ = 0.4321) with the converse for *ABCB1*, high basal expression was associated with decreased sensitivity (*R*^2^ = 0.4249) (Supplementary Figure 2).

### Maintenance of SP-2509 drug resistant state

**Figure 4:**
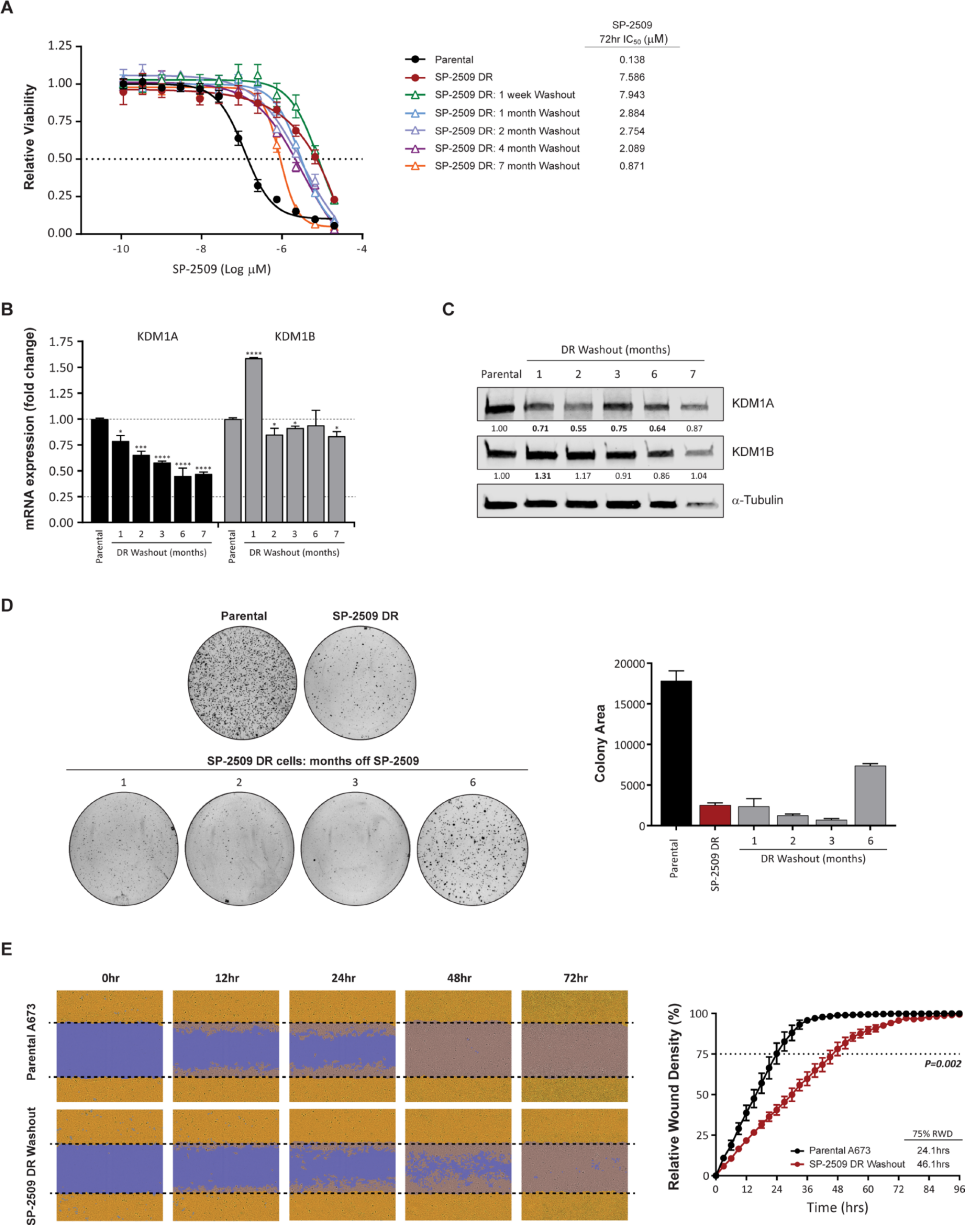
KDM1A is downregulated during SP-2509 washout (**A**) Viability of parental, SP-2509 DR and SP-2509 DR-washout cells (indicated times) following 72 hrs of SP-2509 treatment. Cell viability assessed through CellTiter Glo analysis. Data represents mean ± STDEV from triplicate reactions. Basal KDM1A and KDM1B mRNA (**B**) and protein (**C**) levels of parental and SP-2509 DR-washout cells (months of washout indicated). α-Tubulin was used as a Western blot loading control. (**D**) Representative soft agar anchorage independent growth assays of parental, SP-2509 DR and SP-2509 DR-washout cells. Quantification data represents mean colony area ± STDEV from two plates. (**E**) Representative IncuCyte scratch assay images of parental and SP-2509 DR 5-month washout cells, 0, 12, 24, 48 and 72 hrs post wounding. Quantification of percentage relative wound density (RWD) also depicted. Data represents mean RWD ± SEM from three independent experiments. Asterisks denote statistical significance ^*^P < 0.05, ^***^P < 0.001, ^****^P < 0.0001.

In order to assess whether SP-2509 drug resistance is reversible, SP-2509 treatment was withdrawn from A673 DR cells with cell viability and transformation capacity assessed over a 7 month period. High level resistance to SP-2509 (IC_50_ > 2 μM) was maintained for 4 months, with IC_50_ values dropping to 0.871μM 7-months post withdrawal (Figure 4A), which still equated to a 6.3 fold higher SP-2509 concentration required to reduce viability by 50% compared to parental A673 cells. We previously showed that *KDM1A* but not *KDM1B* mRNA levels were significantly reduced in SP-2509 DR cells (Figure 1F, 1G). Similarly, both KDM1A mRNA and protein levels were significantly reduced (>20%) in SP-2509 DR cells across the entire withdrawal period, with a maximum mRNA reduction of 55.1% observed 6-months post withdrawal. Conversely, a significant increase in KDM1B mRNA and protein levels was observed 1-month post washout out, however these levels returned to near baseline for the remainder of the washout period (Figure 4B, 4C). Furthermore, SP-2509 DR-washout cells were significantly impaired in their ability to form colonies in soft agar, across all time points (Figure 4D). Interestingly, 6 months post washout, these cells regained some anchorage independent growth capacity, with a 65.5% increase in colony area observed compared to SP-2509 DR cells. However, even after 6 months of drug washout, DR cells never regained the full transformation capacity as seen in parental A673 cells (58.7% reduction in colony area compared to parental). To assess the migration ability of SP-2509 DR cells, scratch assays of parental and SP-2509 DR cells 5 months post washout were also conducted. SP-2509 DR-washout cells were significantly impaired in their ability to close the scratch wound by 75% (24.1 versus 46.1 hrs respectively) (*P = 0.002*) (Figure 4E). These results suggest that even after drug withdrawal, DR cells maintain their DR state.

### SP-2509 drug resistant cells exhibit a mesenchymal like morphology

Our RNA-Seq analysis of SP-2509 DR cells revealed a significant enrichment of genes associated with the accumulation of extracellular matrix proteins. As such, microscopic evaluation of SP-2509 DR cells revealed a distinct morphological change compared to parental A673 cells. Parental cells displayed a typical polarized cobblestone like appearance synonymous with epithelial cells, whereas SP-2509 DR cells harbored a mesenchymal spindle-like, fibroblastic morphology indicative of epithelial-mesenchymal (EMT) transition. Indeed, quantification of cell size revealed a 52.2% increase in cell length upon acquisition of SP-2509 DR (*P* < 0.0001) (Supplementary Figure 3A). Interestingly, several studies have reported that Ewing sarcoma cell lines converge towards a mesenchymal/fibroblastic morphology upon EWS/FLI silencing [18, 19]. Surprisingly, this phenotype was rapidly reversible upon drug withdrawal. Within 4 days of SP-2509 removal a 34.5% decrease in cell length between SP-2509 DR and washout cells was observed. Although these 4-day washout cells were still significantly longer in length compared to parental A673 (*P = 0.0005*), a rounded cell morphology was more pronounced. Following 8 days of SP-2509 removal, no significant difference in cell length between parental and SP-2509 DR-washout cells was observed (Supplementary Figure 3A, 3B). To confirm the mesenchymal-like state induced upon SP-2509 DR, 51 markers of EMT were assessed. Compared to parental cells, 24 (47.1%) of these markers were significantly modulated (>1.5 fold change) in SP-2509 DR cells. The majority of genes associated with EMT including *SNAI1*, *MMP3* and *TIMP1* were significantly upregulated upon drug resistance. In addition, genes normally down-regulated during EMT including *SPP1*, *DSP, TSPAN13* and *TFPI2* were also significantly upregulated (Supplementary Figure 3C). Interestingly, increased expression of *Zyxin,* an established EWS/FLI repressed gene [20] which regulates the actin cytoskeleton by aiding in the stabilization of actin stress was also observed.

### SP-2509 DR-washout cells regain sensitivity to vincristine

**Figure 5:**
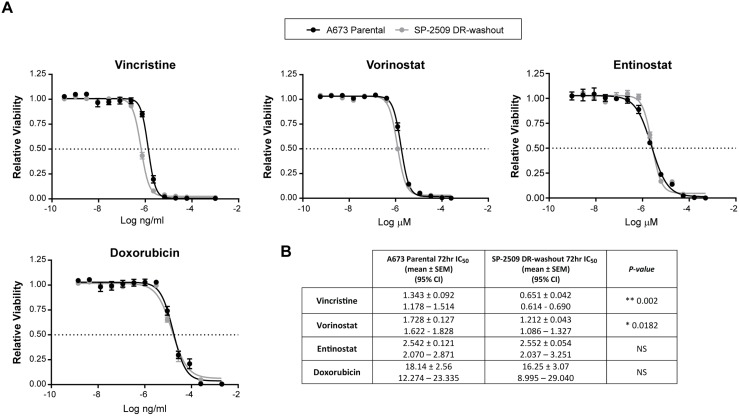
SP-2509 DR-washout cells lose their enhanced sensitivity to HDAC inhibitors (**A**) Parental and SP-2509 DR-washout A673 cells were treated with the indicated agents for 72 hrs. Cell viability was assessed through CellTiter Glo analysis. Data represents mean viability ± SEM from three independent experiments. (**B**) Mean IC_50_ ± SEM and 95% confidence interval for each agent.

To assess whether the chemotherapeutic sensitivity profile of SP-2509 DR cells is maintained following washout, viability assays after treatment with entinostat, doxorubicin, vincristine, and vorinostat were performed. Interestingly, sensitivity to vincristine was regained (IC_50_: Parental 1.343 μM versus DR 0.651 μM) and was significantly lower than parental cells (*P = 0.002*). In contrast, enhanced hypersensitivity to the HDAC inhibitors entinostat (IC_50_: Parental 2.542 μM versus DR 2.552 μM) and vorinostat (IC_50_: Parental 1.728 μM versus DR 1.212 μM) was lost with IC_50_ values comparable to that of parental cells (Figure 5). These results suggest that additional cycles of the traditional Ewing sarcoma chemotherapeutic agent vincristine could be utilized to overcome SP-2509 DR cells.

### Prolonged resistance to SP-2509 is not attributed to a distinct mutational profile

In order to ascertain whether maintenance of SP-2509 DR is driven by a distinct mutational profile, SP-2509 DR-washout cells (3 months) were submitted for WES analysis. To our surprise 43 variants were identified, with none overlapping with the 23 mutations previously reported in our SP-2509 DR cells (Supplementary Table 2). Of these 43 mutations, 32 (74.4%) resulted in non-synonymous single nucleotide variants (SNP), with an A>T transversion in the extended intronic splice region of *EQTN* (Equatorin) occurring in 43.3% of the population. This suggests that all clones present in our original SP-2509 DR population are lost during drug withdrawal. The second and third highest ranking variants (non-synonymous SNP’s) were both detected in *EML4* (Echinoderm Microtubule Associated Protein Like 4), Q872R (32.9%), N873D (32.6%). No mutations in *KDM1A* or its interacting partners (CoREST, NuRD, HDAC) were observed. These results strengthen our previous WES findings that mutation of *KDM1A* does not drive resistance to *KDM1A* blockade.

### SP-2509 DR-washout cells maintain the same transcriptomic profile as SP-2509 DR cells

**Figure 6:**
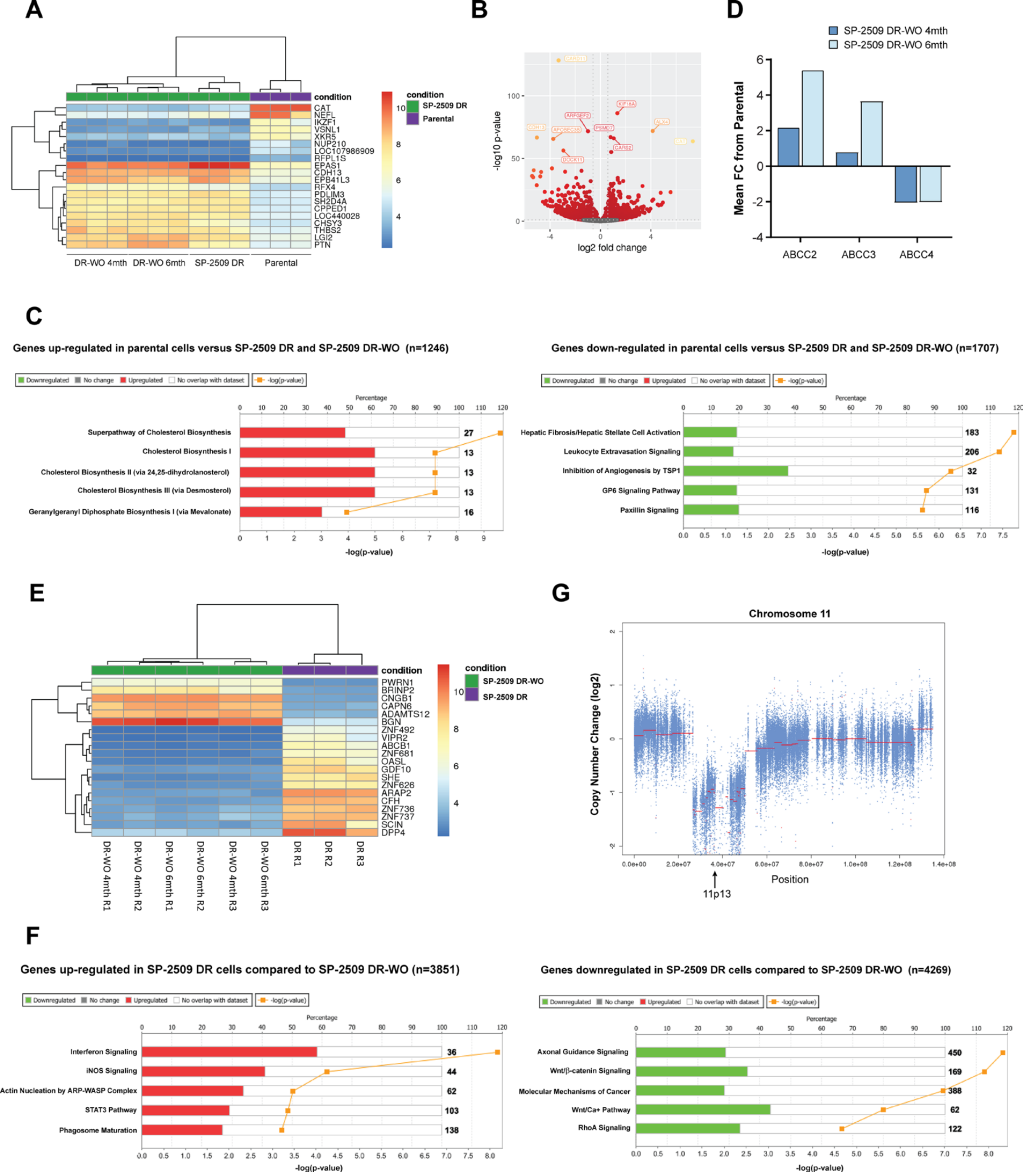
SP-2509 DR and SP-2509 DR-washout cells show the same transcriptional profile (**A**) Unsupervised clustering analysis of the basal transcriptional profile of parental, SP-2509 DR and SP-2509 DR-washout (WO) (4 and 6 months) A673 cells. The top 20 differentially expressed genes from RNA-Seq analysis are shown. (**B**) Volcano plot showing the Log2 fold change of genes that are specifically expressed in parental A673 cells compared to SP-2509 DR lines. The Log_10_ of *P*-value for significance in fold change is plotted on the y-axis. (**C**) IPA of genes significantly up- and down-regulated in parental cells versus all SP-2509 DR cells (>1.5 fold). Percentage gene pathway overlap shown. (**D**) Average normalized expression of *ABCC2*, *ABCC3*, and *ABCC4* in SP-2509 DR-WO cells (4 and 6 months). Data represents fold change from parental A673 cells. (**E**) Unsupervised clustering analysis of the basal transcriptional profile of SP-2509 DR and SP-2509 DR-WO (4 and 6 months) A673 cells. The top 20 differentially expressed genes from RNA-Seq analysis are shown. (**F**) IPA of genes significantly induced/repressed in SP-2509 DR cells compared to SP-2509 DR-WO cells (>1.5 fold). Percentage gene pathway overlap shown. Numbers in bold indicate the number of genes for the specific pathway. (**G**) Copy number analysis of chromosome 11 in SP-2509 DR cells. Approximate position of 11p13, the region encoding catalase is depicted.

To address whether the transcriptomic profile of SP-2509 DR-washout cells reverts back to a parental phenotype, RNA-Seq analysis of SP-2509 DR cells 4 and 6 months post washout was conducted. Unsupervised hierarchical clustering of parental, SP-2509 DR and SP-2509 DR-washout cells revealed that SP-2509 DR-washout cells (4 and 6 months) shared similar transcriptomic profiles as SP-2509 DR cells which was distinct from parental cells (Figure 6A, 6B). This suggests that even following drug withdrawal high and low level DR cells are unable to revert back to their parental phenotype possibly due to fixation into a stochastic epigenetic state. In all, 1246 genes were significantly upregulated in parental cells compared to all SP-2509 DR states with 1707 genes significantly downregulated (>1.5 fold) (Figure 6C). The top 5 genes highly upregulated in parental cells were *CAT*, *NUP210*, *IKZF1*, *RFPL1S* and *VSNL1*, with IPA revealing enrichment for cholesterol (I, II and III) and geranylgeranyl diphosphate biosynthesis pathways. The top 5 genes significantly downregulated in parental cells compared to all SP-2509 DR counterparts were *BMP2*, *ITIH3*, *RFX4*, *CHSY3* and *APC5,* with IPA once again revealing enrichment for the Hepatic Fibrosis pathway in DR cells (*p = 1.72 × 10^8^*). Other pathways included leukocyte extravasation signaling, and inhibition of angiogenesis by TPS1 and GP6 signaling. In addition, enhanced expression of the multi-drug resistance genes, *ABCC2* and *ABCC3*, and reduced expression of *ABCC4* was noted for both washout time points compared to parental cells (Figure 6D).

Upon removal of parental cells from our unsupervised hierarchical clustering analysis, distinct clustering between SP-2509 DR and washout cells was observed (Figure 6E). In comparison to SP-2509 DR-washout cells (4 and 6 months) 3851 and 4269 genes were significantly induced/repressed respectively in SP-2509 DR cells. Compared to washout, IPA of SP-2509 DR cells showed significant enrichment for interferon and iNOS signaling pathways, with washout cells showing enrichment for axonal guidance and Wnt/β catenin signaling (Figure 6F). Interestingly, using a FDR of 0.1 and a fold change of >1.5, no significant difference between high and low level DR cells (6 and 4 months washout respectively) was noted. Together these results highlight that SP-2509 resistance is not mediated through mutation, but through upregulation of multi-drug resistance genes and the generation of a non-reversible transcriptional profile that is maintained even post drug withdrawal.

Consistent across both SP-2509 DR and washout cells, Catalase (*CAT*) was one of the highest ranked genes that was significantly down regulated compared to parental controls. Catalase, located on 11p13, is an important antioxidant enzyme that dismutates hydrogen peroxide into water and molecular oxygen. In addition to its dominant catalytic activity, catalase also decomposes peroxynitrite, and oxidizes nitric oxide to nitrogen. As such, catalase plays an essential role in defending cells against oxidative damage, a key inducer of the endoplasmic reticulum stress response. Several studies have demonstrated that resistance to doxorubicin can be attributed to decreased catalase activity [21–23] with expression predominantly regulated at the transcriptional level by transcription factors that induce or repress the activity of catalase promoters [24]. Within its core promoter the presence of DNA binding sites for transcription factors such as NF-Y, Sp1, FoxO1 and FoxM1 positively regulate the expression of catalase [24]. No significant decrease in the expression of these transcription factors was observed in our SP-2509 DR cell lines. Studies have also shown that decreased catalase expression can be attributed to loss of heterozygosity, deletion of chromosome 11, phosphorylation of Tyr231 and Tyr386 and DNA hypermethylation [24]. Copy number analysis of chromosome 11 revealed a 20MB deletion within the 11p13 region, suggesting that reduced catalase expression in SP-2509 DR cells is due to genetic alteration of this locus (Figure 6G).

### SP-2509 DR cells display altered expression of key transcriptional co-repressors

Given the striking transcriptional similarity between SP-2509 DR and washout cells, we next sought to define the core subset of genes that govern SP-2509 drug resistance. Venn diagram overlap analysis of SP-2509 DR, and SP-2509 DR-washout cells (4 and 6 months) revealed a 16.7% (820 genes) and 14.9% (809 genes) overlap between genes that were significantly up and down-regulated compared to A673 parental cells respectively (Supplementary Figure 4A). Interestingly, when only comparing SP-2509 DR-washout cohorts, a 74.8% (1876 genes) and 74.3% (2025 genes) overlap in up/down-regulated genes was observed, strongly supporting that SP-2509 drug resistance is mediated through acquisition of a fixed non-reversible epigenetic state (Supplementary Figure 4A, 4B). Indeed, principal component analysis clearly demonstrated strong separation between all three conditions (parental, SP-2509 DR and washout), with all SP2509 DR-washout replicates inseparable (Supplementary Figure 4C). Molecular function and biological process analysis of genes universally induced across all drug resistant cell cohorts revealed enrichment for extracellular matrix/structure binding and organization pathways. Surprisingly for repressed genes, cell morphogenesis involved in neuron differentiation/axonogenesis and transcription factor activity were the top biological and molecular pathways respectively (Supplementary Figure 4B).

KDM1A is a key component of various protein complexes that contain transcriptional co-repressors, including the RE1-silencing transcription factor (REST) corepressor CoREST/RCOR, BHC80, HDAC1, HDAC2, CtBP and several zinc finger proteins [16, 25–28]. Indeed, EWS/FLI preferentially recruits transcriptional repressor complexes, such as the nucleosome remodeling and histone deacetylase (NuRD) complex with its associated HDACs and KDM1A, to mediate transcriptional repression of critical EWS/FLI targets [16]. In SP-2509 DR cells, expression analysis of 18 NuRD and RCOR members revealed strong repression in 11/18 components with the strongest repression observed for *GATAD2A* and *HDAC2* (5.9 and 5.3 fold change respectively from parental cells) (Supplementary Figure 4D). Importantly, *RCOR1* and *RCOR2*, core components of the REST complex were also significantly down-regulated. Surprisingly out of the 18 members, only *RCOR1, RCOR2* and *BHC80/PHF21A* were significantly modulated in both SP-2509 DR-washout time points. Similarly to SP-2509 DR cells, *RCOR1* was repressed, however *RCOR2* was significantly upregulated (>2.0 fold increase). Sharing 70% sequence similarity with *RCOR1*, *RCOR2* has a similar biochemical function as *RCOR1* and has been shown to form protein complexes with KDM1A to facilitate its nucleosomal demethylation activity in embryonic stem cells (ESC) [29]. RCOR2 is predominantly expressed in ESCs and the central nervous system, whereas the expression of RCOR1 is more ubiquitous. Moreover, *RCOR2* but not *RCOR1* plays an important role in regulating ESC pluripotency and reprogramming somatic cells to pluripotency and affects neural stem cell proliferation and neurogenesis during cortical development [29, 30].

EWS/FLI interacts with the NuRD complex to mediate transcriptional repression of the Ewing sarcoma tumor suppressor Lysyl oxidase (*LOX*) [16]. Interestingly, this EWS/FLI repressed target was significantly up-regulated (maximum 8.2 fold), across all SP-2509 DR cell populations (Supplementary Figure 4E). Together, these results may suggest that SP-2509 drug resistance is mediated through alteration of key EWS/FLI transcriptional co-repressor complexes leading to epigenetic fixation and cellular differentiation away from the traditional Ewing sarcoma cellular phenotype.

## DISCUSSION

For nearly fifty years, systemic cytotoxic chemotherapy has remained the primary arsenal against Ewing sarcoma. Although proven effective for localized control, a therapeutic clinical plateau has been reached for patients with relapsed and refractory disease. The relatively new paradigm of rationally targeted cancer therapies has dramatically impacted the practice of medical and pediatric oncology, with agents that can target genomically defined vulnerabilities in human tumors validated as effective cancer therapeutics. However, similarly to chemotherapeutic agents, the relatively rapid acquisition of resistance to such treatments significantly limits their utility and remains a substantial challenge in the clinical management of advanced cancers. As such, this study aimed to elucidate mechanisms of resistance to the reversible *KDM1A/LSD1* inhibitor SP-2509. As clinical formulations of SP-2509 (Seclidemstat, *Salarius Pharmaceuticals*), entered phase I clinical testing for Ewing sarcoma patients in 2018, it is imperative that possible mechanisms underlying resistance to this new epigenetic inhibitor are elucidated.

Prolonged chronic exposure of the SP-2509 hypersensitive A673 Ewing sarcoma cell line (IC_50_ < 150 nM) [10] to KDM1A blockade resulted in generation of a SP-2509 drug resistant cell line. Several drug resistant features were revealed, most importantly that resistance to SP-2509 is not mutational in nature as aberrations in *KDM1A* itself were not observed. Indeed, only one non-sense mutation was detected in >50% of the cell population in the mitochondrial ribosomal protein *MRPL45.* Interestingly, *KDM1A* has been shown to coordinate glycolytic metabolism through direct suppression of mitochondrial metabolism genes via H3K4 demethylation [31]. Hence, it may be plausible that disruption of key mitochondrial complexes may drive resistance to SP-2509. However, we believe that resistance is primarily driven through epigenetic avenues. SP-2509 drug resistant lines also displayed major morphological and phenotypic changes including decreased proliferative growth/oncogenic transformation and significant upregulation of genes associated with EMT. Although no synthetic lethality with other drugs was observed, SP-2509 drug resistant cells demonstrated greater sensitivity to epigenetic inhibitors specifically the HDAC agents vorinostat and entinostat. This suggests that HDAC inhibitors could be used to overcome initial SP-2509 drug resistant cell populations.

Following drug washout, high level resistance to SP-2509 (IC_50_ > 2 μM) was maintained for a minimum of 4 months, with residual low level resistant cells displaying the same transcriptomic profile of both SP-2509 drug resistant and high level resistant washout counterparts. Finally, we show that SP-2509 DR and washout cells significantly down-regulate *RCOR1*, a key member of the REST complex required for EWS/FLI mediated transcriptional repression. Together, these findings support the intriguing possibility that resistance to epigenetic inhibitors such as SP-2509 is mediated through epigenetic, and not mutational means.

Several groups have highlighted the emerging role of epigenetic reprogramming in Ewing sarcoma. Indeed, mechanistic studies by our laboratory demonstrated that the NuRD co-repressor complex binds directly to EWS/FLI, and that its associated histone deacetylase and *KDM1A* activities are critical for the repressive function of this oncogenic driver [16]. Inhibition of *KDM1A* either through genetic depletion (shRNA) or small molecule blockade (SP-2509), reverses the EWS/ETS-driven transcriptional signature in Ewing sarcoma. Lastly, EWS/FLI orchestrates large scale epigenetic landscape modifications as evidenced by changes in enhancer marks, histone shifts, chromatin remodeling, super-enhancer status and positioning of other co-regulators such as BAF [2, 3, 32, 33]. Taken together, these studies clearly support that EWS/FLI drives Ewing sarcoma pathogenesis by invoking global deregulation of the epigenome through diverse mechanisms.

The emergence of epigenetic dysregulation and reprogramming in Ewing sarcoma development is likely linked to the biophysical and biochemical properties of the EWS portion of EWS/FLI itself. The EWS portion of EWS/FLI is classified as a low complexity, intrinsically disorder protein [34]. Numerous investigators have defined various characteristics of the EWS transcriptional activation domain, including degenerate hexapeptide repeats (consensus SYGQQS) [1, 34], triplet repeats, and prion like domains [33, 35] which appear to be important for the underlying biophysics of the molecule. It has also been shown that the amino terminal region of EWS and the highly related terminus of FUS undergo unique organizational activities including phase separation and amyloid type fibril formation [33, 36, 37]. Current data suggests that these unique biophysical properties are tied to the functional properties of EWS/FLI with phase separation events allowing inappropriate recruitment of chromatin-remodeling factors which elicit underlying Ewing sarcoma aberrant transcriptional programs [36].

Based on the field's growing appreciation that EWS/FLI mediates epigenetic landscape regulation in Ewing sarcoma, we propose that *KDM1A* is critical for maintaining overall oncogenic competent chromatin configuration and conformation. We suggest a model by which EWS/FLI binds to critical response elements such as GGAA microsatellites which are spread throughout the genome, to mediate large scale changes in chromatin configuration through the self-associating properties of the EWS domain of the fusion protein. We envisage that EWS/FLI physically re-orients chromatin in such a way that new super-enhancers are formed and regions of chromatin are brought into hubs competent for transcriptional regulation, ultimately leading to large scale gene expression changes [36]. We note that EWS/FLI regulates over 4000 target genes [11] directly or indirectly, and as part of this process it is likely that *KDM1A* is critical for establishing and/or maintaining this reprogrammed epigenetic chromatin landscape.

In this model we hypothesize that *KDM1A* inhibition serves to disrupt the chromatin landscape within the cell, ultimately leading to epigenetic reprogramming. Supporting this, we observed that SP-2509 drug resistant cells harbor distinct morphological phenotypes associated with a non-reversible transcriptional profile. Indeed, we propose that when SP-2509 is removed from drug resistant cells, KDM1A again becomes available to help establish and maintain chromatin configuration in conjunction with EWS/FLI. This process is seemingly slow and incomplete as even seven months’ post drug withdrawal, low level resistance to SP-2509 was still apparent. A possible reason for this slow reversal is that cells with newly available uninhibited KDM1A must explore a large epigenetic space stochastically. Chromatin regions, super-enhancers, and changes in epigenetic marks may not be proscriptively organized but rather stochastically explored. Only those epigenetic landscapes that provide evolutionary advantage to a given cell will be maintained and thus allow these drug resistant cells to competitively outcompete their neighbors. Indeed, the role of KDM1A and EWS/FLI in Ewing sarcoma development may involve processes of stochastic epigenetic exploration followed by selection through phenotypic fitness.

Such a model explains a number of poorly understood features in Ewing sarcoma. We and others have noted that the EWS/FLI mediated gene expression profile in Ewing sarcoma cell lines and tumors differs quite significantly from one another [10, 38–40]. Similarly, the transcriptional signature induced by SP-2509 varies considerably. Indeed, only a small subset of genes (103 induced, 82 repressed) were universally modulated across six Ewing sarcoma cell lines following SP-2509 treatment [10]. These diverse expression profiles may explain the significant phenotypic differences observed across Ewing sarcoma cell lines in response to EWS/FLI loss through RNAi interference. In our experience, SK-N-MC cells respond to EWS/FLI depletion through overall cell death with TC32 cells undergoing senescence. In contrast, A673 cells tolerate the loss of EWS/FLI and maintain a near normal proliferative capacity in tissue culture but lose their oncogenic transformation phenotype *in vitro* and *in vivo* [18, 41]. These varying phenotypes and gene expression patterns likely reflect different stochastically developed and subsequently selected chromatin states and gene expression patterns. This model may also explain our inability to fully rescue oncogenic transformation in A673 cells following shRNA knockdown of endogenous EWS/FLI and re-expression through retrovirally introduced cDNA [41]. Using this experimental system, we typically rescue 40% colony formation potential following reintroduction of EWS/FLI. Partial rescue may be attributed to technical challenges such as RNAi mediated off target effects and/or imperfect recovery of EWS/FLI protein levels. An attractive alternative explanation is that perhaps only 40% of the knockdown/rescue population successfully transverse the process of stochastic epigenetic exploration and selection by recovering a fully oncogenic competent chromatin configuration and associated gene expression profile.

In summary, our SP-2509 drug resistance data and proposed speculative model suggests a broad role for epigenetic inhibitors/modulators as cancer therapeutics. We postulate that drug induced epigenetic states are irreversible. At some point Ewing sarcoma cells, like other cancer cells, show an increased stochastic sampling of what is likely an increased availability of epigenetic configurations. Moreover, in Ewing sarcoma, once some of these states are reached, complete reversion is impossible and the cell is trapped working with its newly defined form. We would suggest that any cancer that has a high dependence on epigenetic reprogramming such as pediatric cancers associated with fusion oncoproteins, might be uniquely sensitive to disruption of chromatin state via long term epigenetic inhibition. The critical feature of implementing epigenetic inhibitors into standard treatment backbones is not necessarily treatment at maximum tolerated dose but rather long term exposure to doses sufficient to disrupt a stable and oncogenically competent epigenetic state.

## MATERIALS AND METHODS

### Reagents and cell culture

A673 cells were sourced from American Type Culture Collection (ATCC) and grown in DMEM with L-Glutamine, 10% Fetal Bovine Serum, 1% Penicillin-Streptomycin-Glutamine, 1% Sodium Pyruvate. Cell culture supernatants were tested yearly for Mycoplasma infection using a PCR based detection kit (Southern Biotech, USA) with cells authenticated by STR profiling (Genetica LabCorp, USA). To generate SP-2509 drug resistant cells, A673 cells were exposed to escalating concentrations of SP-2509 for a period of 7 months (100nM increments). SP-2509 was provided by Dr Sunil Sharma (Huntsman Cancer Institute, Utah), Doxorubicin hydrochloride, Etoposide, Vincristine sulfate, and Vorinostat/SAHA were purchased from Cayman Chemical, and Entinostat was purchased from Selleckchem.

### Immunodetection

Whole cell lysate (35 μg) was run on 4–15% Tris-Glycine polyacrylamide gels and transferred onto nitrocellulose membranes using a iBlot2 (Thermo Fisher Scientific) according to the manufacturer's instructions. Membranes were blocked in Odyssey Blocking Buffer (PBS) (LI-COR Bioscience) for 1hr at room temperature. Immunodetection was achieved after incubation with infrared (IR)-dye-conjugated 800CW secondary antibodies (LI-COR) with bands visualized using the Odyssey Imaging System. The following antibodies were used: KDM1A/LSD1 (Cell Signaling, C69G12, 1:1500), KDM1B/LSD2 (Abcam, ab193080, 1:1000), FLI-1 (Abcam, ab15289, 1:500) and α-tubulin (Abcam, ab7291, 1:2000). Densitometry analysis was performed using ImageJ software (V1.51).

### IncuCyte cell proliferation, scratch and caspase 3/7 assays

A673 cells were seeded (4000 cells/well) in 96 clear micro-titer plates (triplicate wells per condition) and left to adhere overnight. For scratch assays 22,000 cells per well were plated in 96 well Essen ImageLock plates with wounds created with the IncuCyte WoundMaker 16 hrs post seeding. If required, escalating concentrations of SP-2509 were added 18 hrs post seeding. Real-time apoptosis assays were assessed through the addition of IncuCyte Caspase-3/7 Apoptosis Assay Reagent (Essen BioSciences), final concentration of 5 μM. Phase contrast and/or green fluorescent images were taken in the IncuCyte ZOOM Kinetic Imaging System (Essen BioScience) at 3 hr intervals for a minimum of 96 hrs. Cell confluence (Phase contrast) or Green Fluorescence (Green Object Count per well) was evaluated using IncuCyte ZOOM 2016A software (Essen BioScience).

### qRT-PCR

Total RNA was extracted using the RNeasy kit (Qiagen) with on-column DNase digestion. cDNA synthesis and subsequent qRT-PCR was performed with 50 ng of total RNA using iTaq Universal SYBR Green 1 Step Reaction Mix (Bio-Rad), according to the manufacturer's protocol. Reaction processing and analysis was performed as previously described [10]. Primer sequences are listed in Pishas *et al*., 2018.

### Soft agar assays

A673 cells were seeded at a density of 7500 cells per 6 cm plate in 0.8% SeaPlaque GTG agarose (Lonza), in media containing 20% FCS, Iscove's modification of Eagle's media, penicillin/streptomycin/glutamine and puromycin. Duplicate plates per condition were seeded. Colonies were quantified using ImageJ software (V1.51) a minimum of 16 days post seeding.

### Viability assays

A673 cells were seeded (6000 cells/well) in 96 white micro-titer plates (triplicate wells per condition) and left to adhere overnight. Cells were treated with vehicle control, media control or serial dilutions of agents (Doxorubicin, Entinostat, Etoposide, SP-2509, Vincristine or Vorinostat) 18 hrs post seeding (0.1% final DMSO concentration). Cell viability was assessed 72 hrs post treatment using CellTiter Glo (Promega) according to the manufacturer's instructions with luminescence read on a GloMax 96 Microplate Luminometer (Promega). Viability was calculated relative to vehicle control cells with IC_50_ values calculated using GraphPad Prism (Version 7.00).

### RNA sequencing

Total RNA was extracted from parental and SP-2509 drug resistant A673 cells, using the RNeasy kit (Qiagen) with on-column DNase digestion and submitted for RNA-Seq analysis (Biomedical Genomics Core, Nationwide Children's Hospital). Sequencing reads from each sample were aligned to the GRCh38.p9 assembly of the Homo Sapiens reference from NCBI using version 2.5.2 b of the splice-aware aligner STAR. Feature coverage counts were calculated with HTSeq, using the GFF file that came with the assembly from NCBI. The default options for feature type, exon, and feature identifier, gene_id, from the GFF were used to identify features for RNA-Seq analysis. Quality control checks for sample preparation and alignment were performed using custom Perl scripts which count types of reads using STAR's mapping quality metric and number of reads aligned to each feature class defined by the feature table that came with the assembly from NCBI. Differential expression analysis was performed using custom R scripts using DESeq2. Significantly differentially expressed features were identified with the criteria of a fold change of absolute value >= 1.5 and an adjusted *p*-value of <= 0.10 (10% FDR). Pathway analysis was conducted using IPA (Qiagen). RNA-seq data is available in the GEO database (http://www.ncbi.nlm.nih.gov/gds) under accession number GSE118871.

### Whole exome sequencing

DNA from parental and SP-2509 drug resistant A673 cells was extracted using the DNeasy Blood and Tissue kit (Qiagen) with RNase A digestion and submitted for WES analysis (Genomic's core, Nationwide Children's Hospital). WES libraries were captured with the Agilent SureSelect Clinical Research Exome kit (Agilent Technologies) and paired-end 151-bp reads were sequenced on the Illumina HiSeq 4000. Average sequencing coverage depth for the samples was 249× (range 227× – 282×). Sequence alignment, post alignment processing, variant calling and genotyping were performed with the Churchill pipeline, resulting in SNPs, INDELs and regions of copy number alteration and loss of heterozygosity using reference build GRCh37. Variant calling specifically was performed on each sample for typical variant discovery, and also in a somatic manner in order to extract differences between the relevant pairs of samples. The results were annotated with gene, transcript, variant effect, and numerous cancer-related databases including frequency in various cancer populations and disease association using SnpEff and custom in-house scripts.

### Statistical analysis

*P* values were calculated using Student *t*-test in Graph Pad Prism (Version 7).

## SUPPLEMENTARY MATERIALS FIGURES AND TABLES






