# Supplementary material for: Ewing sarcoma resistance to SP-2509 is not mediated through KDM1A/LSD1 mutation
Source: Oncotarget. 2018 Nov 23;9(92):36413–29. doi: 10.18632/oncotarget.26326 (PMC6284858; doi:10.18632/oncotarget.26326)
Supplement: Supplementary file 1 [file oncotarget-09-36413-s001.pdf]

Ewing sarcoma resistance to SP-2509 is not mediated through KDM1A/LSD1 mutation

SUPPLEMENTARY MATERIALS

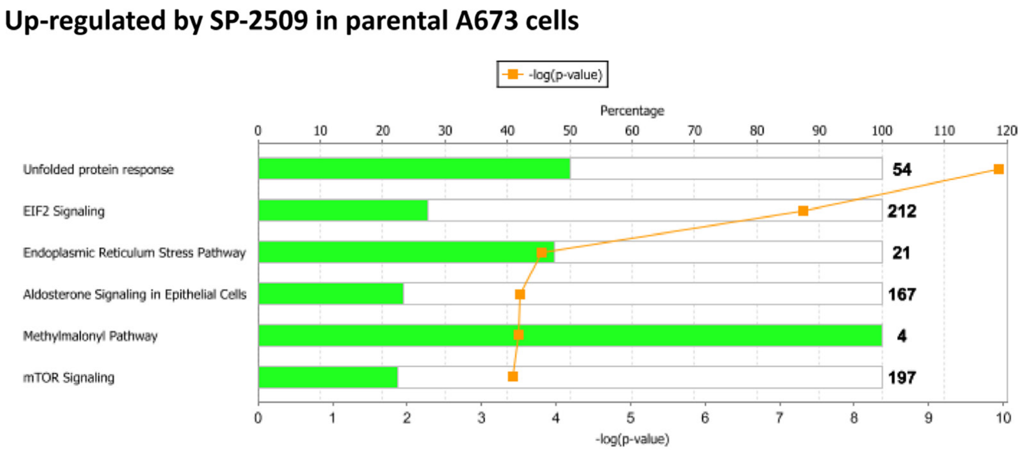

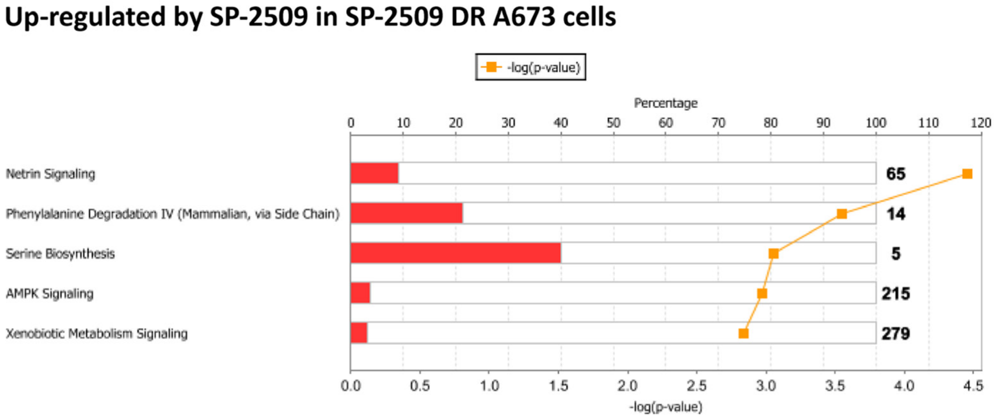

Supplementary Figure 1: SP-2509 drug resistant cells do not engage the endoplasmic reticulum stress response following treatment with SP-2509. RNA-Seq IPA of genes significantly upregulated (>1.5 fold) in parental and SP-2509 drug resistant (DR) cells following treatment with SP-2509 (2 μM, 48 hrs). The percentage gene pathway overlap is shown. Bold numbers indicate the number of genes in that specific pathway.

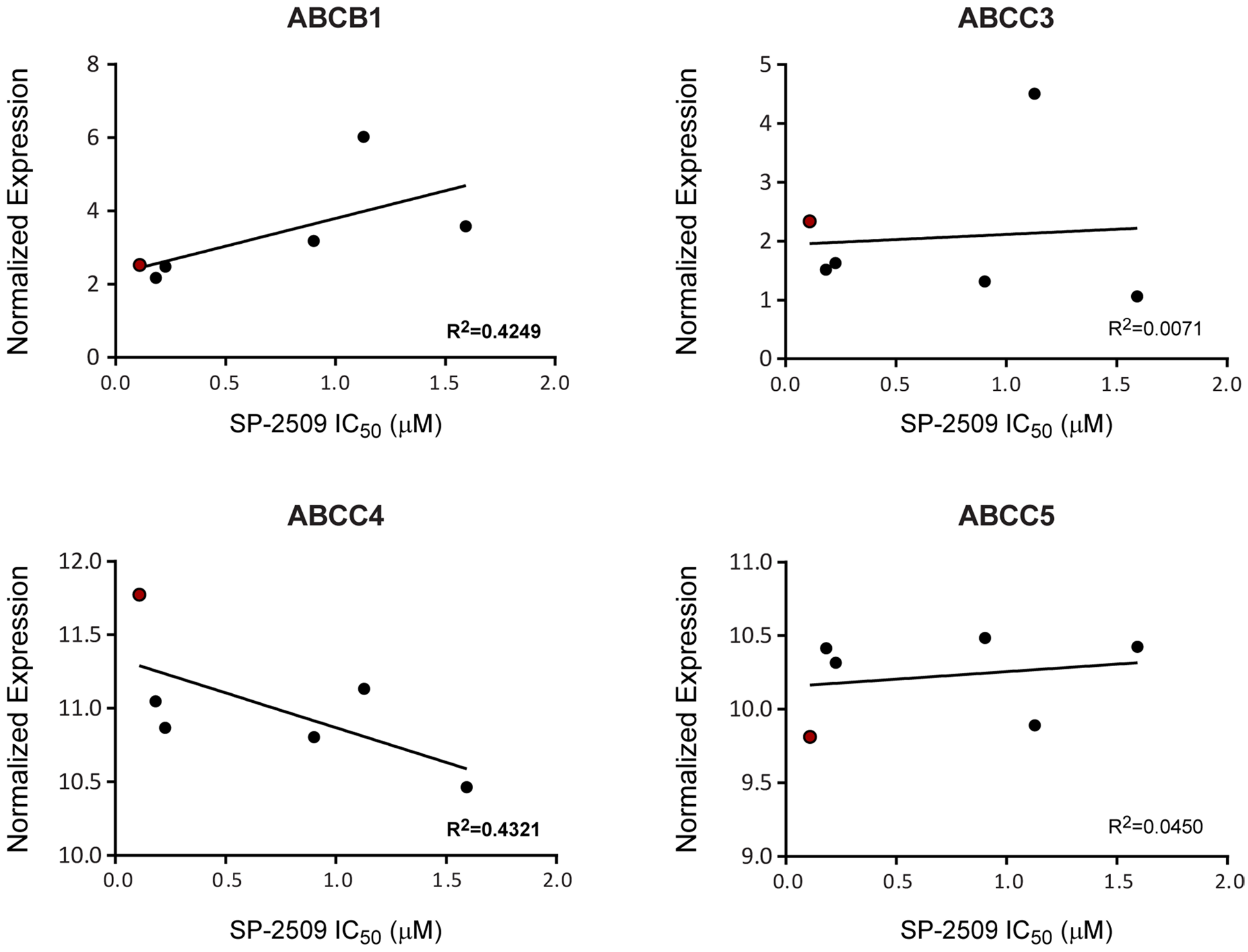

Supplementary Figure 2: Expression of *ABCB1* and *ABCC4* correlate with sensitivity to SP-2509. Correlation between sensitivity to SP-2509 and expression of multi-drug resistance genes. Ewing sarcoma cell line ($n = 6$) SP-2509 sensitivity determined through CellTiter Glo analysis, 72 hrs post treatment. Data taken from Pishas *et al*., 2018. Normalized expression of *ABCB1*, *ABCC3*, *ABCC4* and *ABCC5* taken from RNA-Seq analysis. ● Denotes A673 cells.

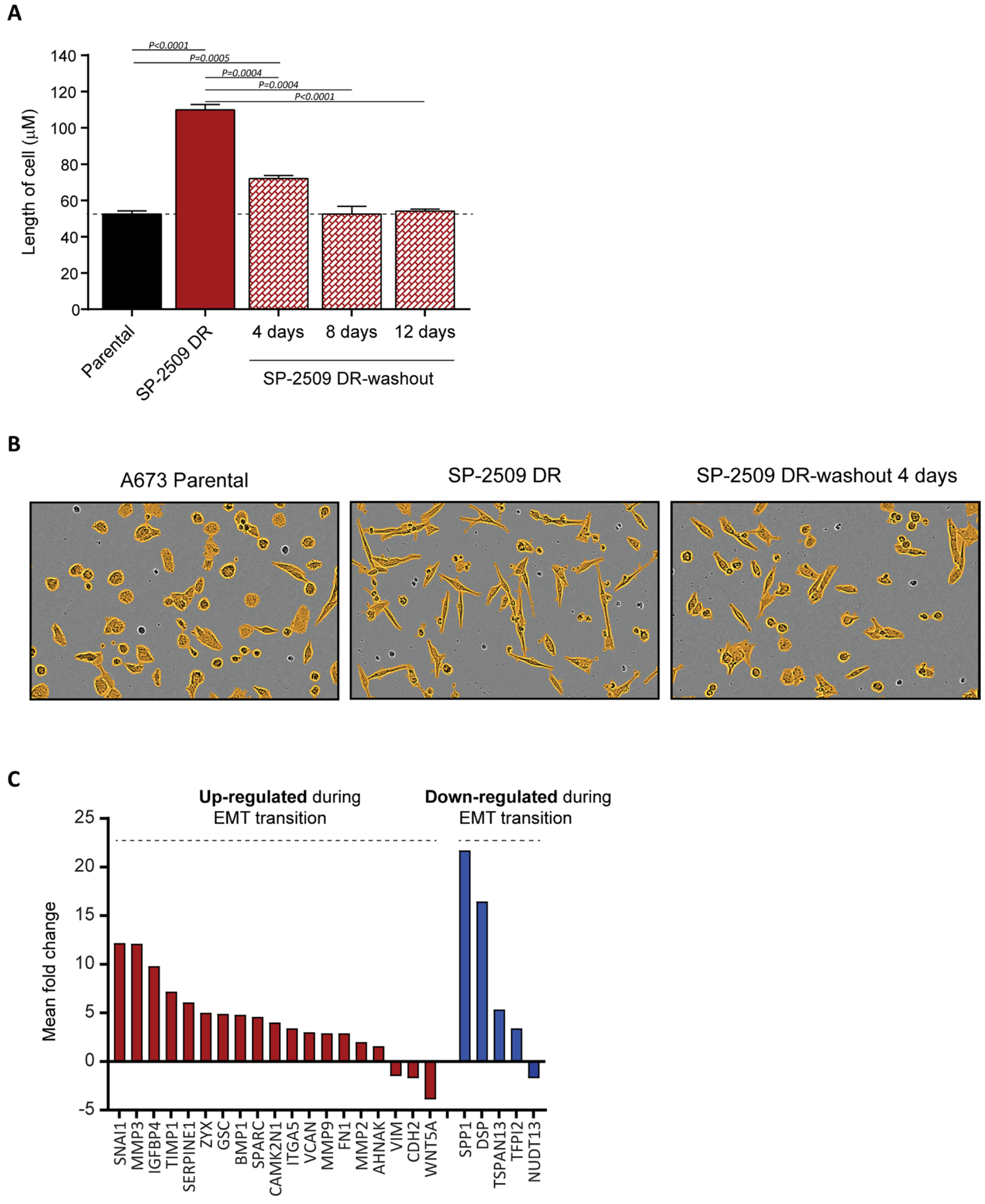

Supplementary Figure 3: SP-2509 drug resistant cells display a reversible EMT morphology. (**A**) Quantification of cell length in A673 parental, SP-2509 drug resistant (DR) and SP-2509 DR cells post washout for the indicated times. Data represents mean length ± SEM from triplicate wells. 10 cells per well, 18 hrs post seeding were analyzed. (**B**) Representative phase contrast IncuCyte Zoom images of parental and SP-2509 DR cells 18 hrs post seeding. (**C**) RNA-Seq expression levels of EMT (epithelial to mesenchymal transition) genes significantly (>1.5 fold) altered in SP-2509 DR cells compared to parental. Data represents mean fold change from parental cells.

A

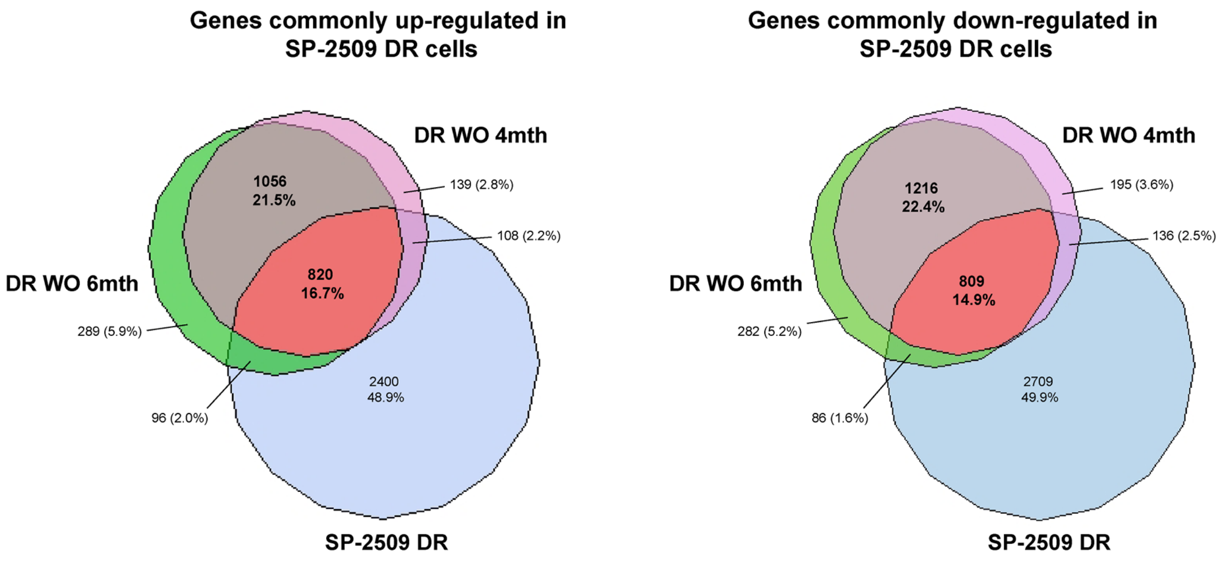

B

Genes commonly up-regulated in SP-2509 DR cells

GO: Biological Process

Rank	ID	Name	p-Value	Bonferroni	Genes from input	Genes in Annotation
1	GO:0030196	Extracellular matrix organization	1.296×10^{-10}	8.090×10^{-7}	40	354
2	GO:0043062	Extracellular structure organization	1.412×10^{-10}	8.815×10^{-7}	40	355
3	GO:0048646	Structure formation involved in morphogenesis	4.082×10^{-10}	2.548×10^{-6}	91	1299
4	GO:0001525	Angiogenesis	4.328×10^{-10}	2.702×10^{-6}	46	464

GO: Molecular Function

Rank	ID	Name	p-Value	Bonferroni	Genes from input	Genes in Annotation
1	GO:0050840	Extracellular matrix binding	4.957×10^{-7}	7.524×10^{-4}	12	56
2	GO:0019838	Growth factor binding	3.021×10^{-6}	4.587×10^{-3}	18	142
3	GO:0005518	Collagen binding	8.107×10^{-6}	1.231×10^{-2}	12	72
4	GO:0005102	Signaling receptor binding	1.498×10^{-5}	2.275×10^{-2}	89	1601

Genes commonly down-regulated in SP-2509 DR cells

GO: Biological Process

Rank	ID	Name	p-Value	Bonferroni	Genes from input	Genes in Annotation
1	GO:0048667	Cell morphogenesis involved in neuron differentiation	5.396×10^{-8}	3.148×10^{-4}	50	590
2	GO:0007409	Axonogenesis	5.426×10^{-7}	3.165×10^{-3}	41	475
3	GO:0048812	Neuron projection morphogenesis	9.523×10^{-7}	5.556×10^{-3}	49	630
4	GO:0000904	Cell morphogenesis involved in differentiation	9.977×10^{-7}	5.820×10^{-3}	60	840

GO: Molecular Function

Rank	ID	Name	p-Value	Bonferroni	Genes from input	Genes in Annotation
1	GO:0000982	Transcription factor activity, RNA polymerase II proximal promoter sequence-specific DNA binding	6.278×10^{-6}	8.946×10^{-3}	32	365
2	GO:0044212	Transcription regulatory region DNA binding	7.673×10^{-6}	1.093×10^{-2}	58	860
3	GO:0000976	Transcription regulatory region sequence-specific DNA binding	8.387×10^{-6}	1.195×10^{-2}	50	704
4	GO:0001067	Regulatory region nucleic acid binding	9.441×10^{-6}	1.345×10^{-2}	58	866

C

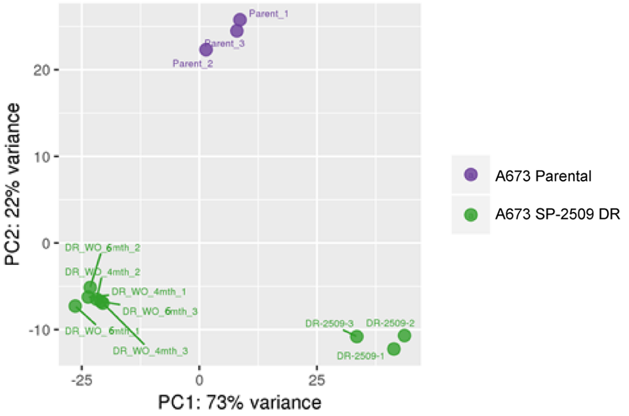

D

NuRD/REST component	Fold change in expression from parental A673		
	SP-2509 DR	SP-2509 DR-WO 4mth	SP-2509 DR-WO 6mth
BHC80/PHF21A	-5.2	-2.2	-2.1
CHD3	-1.6		
CHD4	-1.9		
GATAD2A	-5.9		
GATAD2B			
HDAC1			
HDAC2	-5.3		
MBD2			
MBD3			
MTA1	-5.0		
MTA2	-1.6		
MTA3			
RBBP4	-2.1		
RBBP7			
**RCOR1/CoREST1**	-3.5	-2.3	-2
**RCOR2/CoREST1**	-4.1	2.1	2.5
SIN3A	-4.6		
SIN3B	-3.2		

E

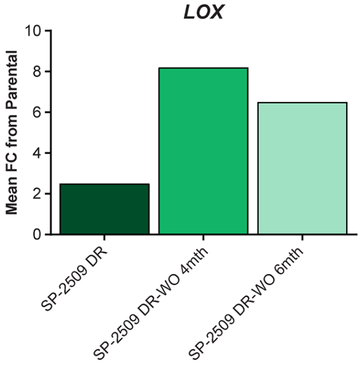

Supplementary Figure 4: Core SP-2509 drug resistant transcriptional profile. (**A**) Venn diagram analysis of RNA-Seq genes universally up/down-regulated (≥1.5 fold change) in SP-2509 drug resistant (DR) and SP-2509 DR-washout (WO) cells (4 and 6 months) compared to parental A673 cells. (**B**) ToppGene Suite analysis of GO biological and molecular function pathways associated with core SP-2509 DR genes (n = 820 up-regulated and n = 809 down-regulated). (**C**) PCA analysis of all SP-2509 DR groups. (**D**) Expression levels of NuRD/REST components in SP-2509 DR cells. Data expressed as normalized fold change (FC) from parental A673 cells. (**E**) Expression levels of *LOX* in SP-2509 DR cells. Data expressed as normalized FC from parental A673 cells.

Supplementary Table 1: Mutations acquired during prolong passage of A673 cells. See Supplementary_Table_1

Supplementary Table 2: Mutations identified in SP-2509 DR washout cells. See Supplementary_Table_2
