# Supplementary material for: Ewing sarcoma resistance to SP-2509 is not mediated through KDM1A/LSD1 mutation
Source: Oncotarget. 2018 Nov 23;9(92):36413–29. doi: 10.18632/oncotarget.26326 (PMC6284858; doi:10.18632/oncotarget.26326)
Supplement: Supplementary file 2 [file oncotarget-09-36413-s002.pdf]

Supplementary Table 1: *Mutations acquired during prolong passage of A673 cells*

Rank	Position	Type	Gene		Loc In Gene	Effect	HGVS	Population %
1	17q21.2	SNP	KAT2A	Lysine Acetyltransferase 2A	CS	Stopgain	p.Tyr807*	40.3%
2 #	11q23.3	SNP	C2CD2L	C2CD2 Like	CS	NS-SNV	p.Glu178Asp	30.5%
3	5q33.3	SNP	CYFIP2	Cytoplasmic FMR1 Interacting Protein 2	CS	NS-SNV	p.Ala437Gly	29.3%
4	5q23.1	SNP	SEMA6A	Semaphorin 6A	CS	NS-SNV	p.Lys640Arg	27.3%
5	22q13.2	SNP	TTLL12	Tubulin Tyrosine Ligase Like 12	splice_site		c.178-1G>T	25.8%
6	17p11.2	SNP	RNF112	Ring Finger Protein 112	CS	NS-SNV	p.Asn156Ile	24.8%
7	1p36.33	SNP	VWA1	Von Willebrand Factor A Domain Containing 1	CS	NS-SNV	p.Cys420Tyr	23.1%
8	4q25	SNP	LRIT3	Leucine Rich Repeat, Ig-Like And Transmembrane Domains 3	CS	NS-SNV	p.Ala514Glu	23.1%
9	8q13.3	SNP	TRPA1	Transient Receptor Potential Cation Channel Subfamily A Member 1	CS	NS-SNV	p.Leu697Pro	22.6%
10	7q32.1	SNP	FSCN3	Fascin Actin-Bundling Protein 3	splice_site		c.145-1G>T	22.1%
11	1q21.3	SNP	TDRD10	Tudor Domain Containing 10	CS	NS-SNV	p.Glu166Asp	21.6%
12	6q16.1	SNP	MMS22L	MMS22 Like, DNA Repair Protein	CS	NS-SNV	p.Arg1212Ser	21.1%
13	20q11.23	SNP	RALGAPB	Ral GTPase Activating Protein Non-Catalytic Beta Subunit	CS	NS-SNV	p.Lys933Glu	21.0%
14	10q24.1	SNP	SLIT1	Slit Guidance Ligand 1	CS	NS-SNV	p.Ala1234Pro	21.0%
15	7q31.1	SNP	LAMB4	Laminin Subunit Beta 4	CS	NS-SNV	p.Leu931Met	20.3%
16	22q13.2	SNP	CHADL	Chondroadherin Like	CS	NS-SNV	p.Leu472Arg	20.1%
17	6q16.3	SNP	ASCC3	Activating Signal Cointegrator 1 Complex Subunit 3	CS	Stopgain	p.Glu156*	19.9%
18	Xp22.2	SNP	GPR143	G Protein-Coupled Receptor 143	CS	NS-SNV	p.Arg237Gly	19.8%
19	17p13.2	SNP	SLC52A1	Solute Carrier Family 52 Member 1	CS	NS-SNV	p.Ala122Glu	19.4%
20	1p34.3	SNP	ZMYM1	Zinc Finger MYM-Type Containing 1	CS	NS-SNV	p.Lys734Asn	19.4%
21	10q26.13	SNP	DMBT1	Deleted In Malignant Brain Tumors 1	CS	NS-SNV	p.Val486Leu	19.3%
22	14q23.2	SNP	ESR2	Estrogen Receptor 2	CS	NS-SNV	p.Val269Ala	19.2%
23	3p25.1	SNP	NUP210	Nucleoporin 210	CS	NS-SNV	p.Gly1269Val	18.8%
24	12p13.2	SNP	CLEC12A	C-Type Lectin Domain Family 12 Member A	splice_region	Stopgain	p.Glu127*	18.8%
25	5p15.2	SNP	CTNND2	Catenin Delta 2	CS	Stopgain	p.Ser39*	18.3%
26	1q31.3	SNP	ASPM	Abnormal Spindle Microtubule Assembly	CS	NS-SNV	p.Lys1603Glu	18.2%
27	9q31.2	SNP	KLF4	Kruppel Like Factor 4	EISR		c.1264+8G>T	18.2%
28	11p15.4	SNP	TRIM66	Tripartite Motif Containing 66	CS	NS-SNV	p.Glu1107Gln	18.1%
29	13q22.1	SNP	KLF12	Kruppel Like Factor 12	CS	NS-SNV	p.Arg396Cys	18.0%

Supplementary Table S1 Continued

30	15q26.3	SNP	ADAMTS17	ADAM Metallopeptidase With Thrombospondin Type 1 Motif 17	CS	NS-SNV	p.Pro639Thr	17.9%
31	19p12	SNP	ZNF493	Zinc Finger Protein 493	CS	NS-SNV	p.Ser23Arg	17.7%
32	7q21.13	DEL	CDK14	Cyclin Dependent Kinase 14	CS	Frameshift	p.Asp319fs	17.6%
33	2p16.3	SNP	NRXN1	Neurexin 1	CS	NS-SNV	p.Glu389Asp	17.4%
34	19p13.3	SNP	ONECUT3	One Cut Homeobox 3	CS	NS-SNV	p.Ala150Pro	17.2%
35	19p13.3	SNP	ZNF556	Zinc Finger Protein 556	CS	NS-SNV	p.Glu63Asp	17.1%
36	17p13.1	SNP	PFAS	Phosphoribosylformylglycinamidine Synthase	CS	NS-SNV	p.Arg1310Leu	17.1%
37	2q12.1	SNP	SLC9A2	Solute Carrier Family 9 Member A2	EISR		c.1515+4G>T	15.9%
38	19q13.12	SNP	ZFP14	ZFP14 Zinc Finger Protein	CS	NS-SNV	p.His190Tyr	15.9%
39	Xp11.22	SNP	FGD1	FYVE, RhoGEF And PH Domain Containing 1	CS	NS-SNV	p.Glu590Asp	15.8%
40	7q21.2	SNP	SAMD9L	Sterile Alpha Motif Domain Containing 9 Like	CS	NS-SNV	p.Ile614Thr	15.6%
41	19q13.2	SNP	CATSPERG	Cation Channel Sperm Associated Auxiliary Subunit Gamma	CS	NS-SNV	p.Ser1036Arg	15.1%
42	15q15.1	SNP	PLCB2	Phospholipase C Beta 2	CS	NS-SNV	p.Glu275Asp	14.7%
43	7q31.1	SNP	LAMB1	Laminin Subunit Beta 1	CS	Stopgain	p.Cys1165*	14.7%
44	6q13	SNP	RIMS1	Regulating Synaptic Membrane Exocytosis 1	CS	NS-SNV	p.Glu1409Lys	13.5%
45	8q24.13	SNP	ANXA13	Annexin A13	CS	NS-SNV	p.Gln12Arg	13.4%
46	17q25.1	SNP	SDK2	Sidekick Cell Adhesion Molecule 2	CS	NS-SNV	p.Pro1750Leu	10.9%
47	7q22.1	SNP	MUC12	Mucin 12, Cell Surface Associated	CS	NS-SNV	p.Thr3456Met	9.0%
48	3p21.31	SNP	XCR1	X-C Motif Chemokine Receptor 1	CS	NS-SNV	p.Val301Ala	7.1%
49	12q24.22	SNP	NOS1	Nitric Oxide Synthase 1	CS	NS-SNV	p.Gly219Glu	6.5%
50	11q12.1	DEL	ZDHHC5	Zinc Finger DHHC-Type Containing 5	EISR		c.753-13_753-10delTCTC	6.3%
51	10q24.33	SNP	PCGF6	Polycomb Group Ring Finger 6	CS	NS-SNV	p.Glu42Ala	5.3%

CS: Coding sequence, EISR: Extended intronic splice region, NS-SNV: Non-synonymous single nucleotide variant, SNP: Single Nucleotide Polymorphism

C2CD2L mutation also observed in SP-2509 drug resistant cells
