# Supplementary material for: Ewing sarcoma resistance to SP-2509 is not mediated through KDM1A/LSD1 mutation
Source: Oncotarget. 2018 Nov 23;9(92):36413–29. doi: 10.18632/oncotarget.26326 (PMC6284858; doi:10.18632/oncotarget.26326)
Supplement: Supplementary file 3 [file oncotarget-09-36413-s003.pdf]

Supplementary Table 2: *Mutations identified in SP-2509 DR washout cells*

Rank	Type	Position	Gene		Loc In Gene	Effect	HGVS	Population	Present in DR cells
1	SNP	9p21.2	EQTN	Equatorin	EISR			43.3%	No
2	SNP	2p21	EML4	Echinoderm Microtubule Associated Protein Like 4	CS	NS-SNV	p.Gln872Arg	32.9%	No
3	SNP	2p21	EML4	Echinoderm Microtubule Associated Protein Like 4	CS	NS-SNV	p.Asn873Asp	32.6%	No
4	SNP	14q21.3	POLE2	DNA Polymerase Epsilon 2, Accessory Subunit	CS	NS-SNV	p.Arg9Gln	31.0%	No
5	SNP	6q24.3	ADGB	Androglobin	CS	NS-SNV	p.Leu1363Trp	31.0%	No
6	SNP	13q12.13	RNF6	Ring Finger Protein 6	CS	NS-SNV	p.Ser209Gly	30.1%	No
7	SNP	8q11.23	RB1CC1	RB1 Inducible Coiled-Coil 1	CS	NS-SNV	p.Ala445Glu	29.4%	No
8	SNP	9q34.12	ABL1	ABL Proto-Oncogene 1, Non-Receptor Tyrosine	CS	NS-SNV	p.Gly864Val	29.1%	No
9	SNP	6q27	C6orf118	Chromosome 6 Open Reading Frame 118	CS	NS-SNV	p.Leu182Met	28.4%	No
10	SNP	13q12.13	WASF3	WAS Protein Family Member 3	CS	NS-SNV	p.Glu462Asp	27.4%	No
11	SNP	19q13.41	NKG7	Natural Killer Cell Granule Protein 7	CS	NS-SNV	p.Gln124His	27.3%	No
12	SNP	5q22.3	CCDC112	Coiled-Coil Domain Containing 112	CS	Stopgain	p.Lys87*	26.2%	No
13	SNP	Xq22.3	NRK	Nik Related Kinase	CS	NS-SNV	p.Ser927Arg	26.1%	No
14	SNP	3p22.3	CRTAP	Cartilage Associated Protein	CS	NS-SNV	p.Arg6Gln	25.8%	No
15	DEL	8p21.2	TRIM35	Tripartite Motif Containing 35	EISR			25.2%	No
16	SNP	3p25.1	FGD5	FYVE, RhoGEF And PH Domain Containing 5	splice_site			24.2%	No
17	SNP	16q22.1	LRRC29	Leucine Rich Repeat Containing 29	CS	NS-SNV	p.Gln146Lys	24.1%	No
18	SNP	Xq25	LOC100129520		CS	NS-SNV	p.Trp91Leu	23.8%	No
19	SNP	1p13.1	PTGFRN	Prostaglandin F2 Receptor Inhibitor	CS	NS-SNV	p.His341Gln	23.8%	No
20	SNP	11p15.1	OTOG	Otogelin	CS	NS-SNV	p.Gly859Arg	23.8%	No
21	SNP	1q25.3	CACNA1E	Calcium Voltage-Gated Channel Subunit Alpha1 E	CS	NS-SNV	p.Phe651Leu	23.4%	No
22	SNP	7q22.1	CYP3A4	Cytochrome P450 Family 3 Subfamily A Member 4	CS	NS-SNV	p.Leu132Phe	22.7%	No
23	SNP	1q32.1	NFASC	Neurofascin	CS	NS-SNV	p.Gly232Arg	22.3%	No
24	DEL	14q32.33	AHNAK2	AHNAK Nucleoprotein 2	CS	Non-	p.Leu819_Asp823del	21.8%	No
25	SNP	10q23.31	STAMBPL1	STAM Binding Protein Like 1	CS	NS-SNV	p.Val334Ala	21.2%	No
26	SNP	16p12.2	OTOA	Otoancorin	CS	NS-SNV	p.Leu309Met	21.1%	No
27	SNP	16p13.3	TPSD1	Tryptase Delta 1	EISR			21.1%	No
28	SNP	19p13.2	MUC16	Mucin 16, Cell Surface Associated	CS	NS-SNV	p.Pro7627His	20.7%	No
29	SNP	1p13.3	ATXN7L2	Ataxin 7 Like 2	CS	NS-SNV	p.Val18Leu	19.1%	No
30	SNP	1q21.1	NBPF10	NBPF Member 10	CS	NS-SNV	p.Ser319Ala	18.8%	No
31	SNP	4q31.3	TMEM131L	Transmembrane 131 Like	CS	NS-SNV	p.Asp1192Asn	18.2%	No
32	SNP	20p11.21	CST9	Cystatin 9	CS	Stoploss		16.9%	No
33	SNP	Xp11.22	DGKK	Diacylglycerol Kinase Kappa	CS	NS-SNV	p.Gly1125Ala	15.6%	No
34	SNP	2q12.3	RGPD4	RANBP2-Like And GRIP Domain Containing 4	CS	NS-SNV	p.Glu1628Gln	15.4%	No
35	SNP	19p13.3	ATP8B3	ATPase Phospholipid Transporting 8B3	CS	NS-SNV	p.Met314Leu	14.8%	No
36	SNP	7q11.23	CLDN4	Claudin 4	CS	startloss		12.4%	No
37	SNP	Xp21.3	MAGEB10	MAGE Family Member B10	CS	NS-SNV	p.Asp112Glu	12.1%	No
38	INS	4q31.3	PRSS48	Protease, Serine 48	CS	Frameshift	p.Gln42fs	11.9%	No
39	SNP	6p24.2	ELOVL2	ELOVL Fatty Acid Elongase 2	CS	Stopgain	p.Ser239*	11.3%	No
40	SNP	10q22.1	PRF1	Perforin 1	CS	NS-SNV	p.Gly426Asp	9.5%	No
41	SNP	3q26.2	TNIK	TRAF2 And NCK Interacting Kinase	CS	NS-SNV	p.Arg912His	9.5%	No
42	SNP	12p13.2	YBX3	Y-Box Binding Protein 3	CS	NS-SNV	p.Asn257Lys	7.8%	No
43	SNP	16p11.2	SULT1A1	Sulfotransferase Family 1A Member 1	EISR			5.9%	No

CS: Coding sequence, DR: Drug resistant, EISR: Extended intronic splice region, NS-SNV: Non-synonymous single
